# Updated catalogue and taxonomic notes on the Old-World scorpion genus *Buthus* Leach, 1815 (Scorpiones, Buthidae)

**DOI:** 10.3897/zookeys.686.12206

**Published:** 2017-07-24

**Authors:** Pedro Sousa, Miquel A. Arnedo, D. James Harris

**Affiliations:** 1 CIBIO Research Centre in Biodiversity and Genetic Resources, InBIO, Universidade do Porto, Campus Agrário de Vairão, Vairão, Portugal; 2 Departamento de Biologia, Faculdade de Ciências da Universidade do Porto, Porto, Portugal; 3 Department of Evolutionary Biology, Ecology and Environmental Sciences, and Biodiversity Research Institute (IRBio), Universitat de Barcelona, Barcelona, Spain

**Keywords:** Taxonomy, new synonymy, new combination, new status, Geographic distribution, Africa, Asia, Europe, diagnostic characters

## Abstract

Since the publication of the ground-breaking “Catalogue of the scorpions of the world (1758–1998)” (Fet et al. 2000) the number of species in the scorpion genus *Buthus* Leach, 1815 has increased 10-fold, and this genus is now the fourth largest within the Buthidae, with 52 valid named species. Here we revise and update the available information regarding *Buthus*. A new combination is proposed: *Buthus halius* (C. L. Koch, 1839), **comb. n.** from Portugal and Spain. *B. halius* is removed from junior synonymy with *Buthus occitanus* (Amoreux, 1789), and proposed as a senior synonym of *B. ibericus* Lourenço & Vachon, 2004, **syn. n.** Moreover, following I.C.Z.N. article 23.9.2 we propose to maintain as valid *B. ibericus* **(*nomen protectum*)** and to consider the disued *B. halius* as a ***nomen oblitum***. *Buthus europaeus tridentatus* Franganillo, 1918 is proposed as a junior synonym of *B. occitanus* (Amoreux, 1789), **syn. n.** *Buthus sabulicola* Touloun, 2012 is proposed as a junior synonym of *Buthus bonito* Lourenço & Geniez, 2005, **syn. n.** *Buthus occitanus tunetanus neeli* Gysin, 1969 is proposed as an informal senior synonym of *Buthus tassili* Lourenço, 2002, **informal syn. n.** Two taxa are rised to species rank, *Buthus nigrovesiculosus* Hirst, 1925, **stat. n.** and *Buthus parroti* Vachon, 1949, **stat. n.**. We further confirm the restricted distribution of *B. occitanus* that is confined to southeastern France and northwestern Iberian Peninsula and does not occur in North Africa. Additionally, *Androctonus barbouri* (Werner, 1932), **comb. n.** from the Agadir region of Morocco, is hereby transferred to the genus *Androctonus*. We summarize and provide a critical appraisal of the diagnostic characters currently in use for the genus. The catalogue section considers the names for species, subspecies and varieties that have been used for *Buthus* scorpions. Information about types, including collection numbers and localities are included when available. Finally, an annotated listing of synonymies and an updated bibliography are given.

## Introduction

**Figure 1.**
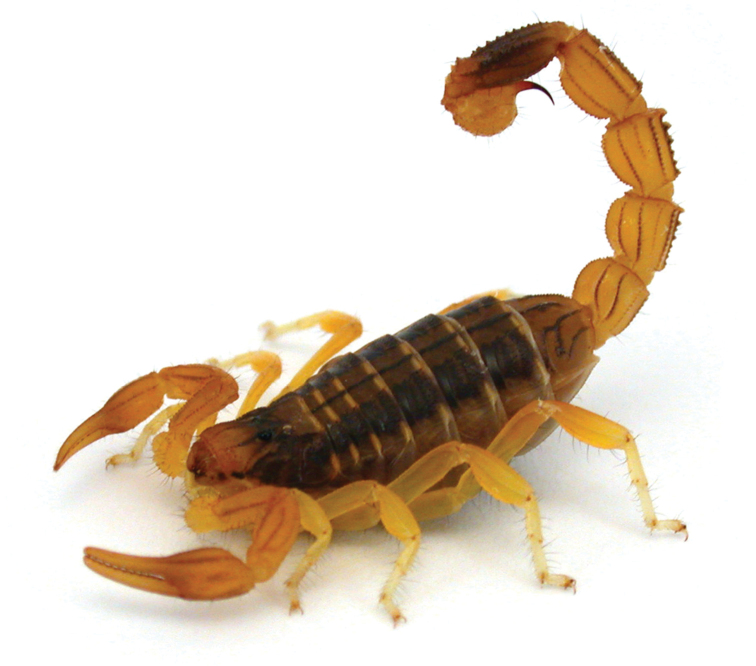
*Buthus mariefranceae*, from south of Morocco. Photo by Arie van der Meijden.

Members of the genus *Buthus* Leach, 1815 are medium-sized scorpions, usually yellowish in colour, with a robust metasoma that ends in a telson with a globular vesicle and a curved aculeus (Fig. 1). *Buthus* rest during the day in burrows under stones or shrubs and are active from dusk till dawn, although their activity typically peaks at the beginning of the night (Cloudsley-Thompson 1956). They are successful scorpions that, when present, tend to be the most abundant scorpion in their habitat, as for example in the Iberian Peninsula and Morocco. *Buthus* are usually described as sit-and-wait predators, although they can also actively search for prey (Skutelsky 1995, Piñero et al. 2013). *Buthus* is among the most venomous of all scorpion genera (Chippaux and Goyffon 2008). *Buthus* venom toxicity is considered much lower in Europe than in North Africa, which can be empirically corroborated by the few severe cases of scorpionism reported for Western Europe when compared to the North African countries (Chippaux and Goyffon 2008).

**Figure 2.**
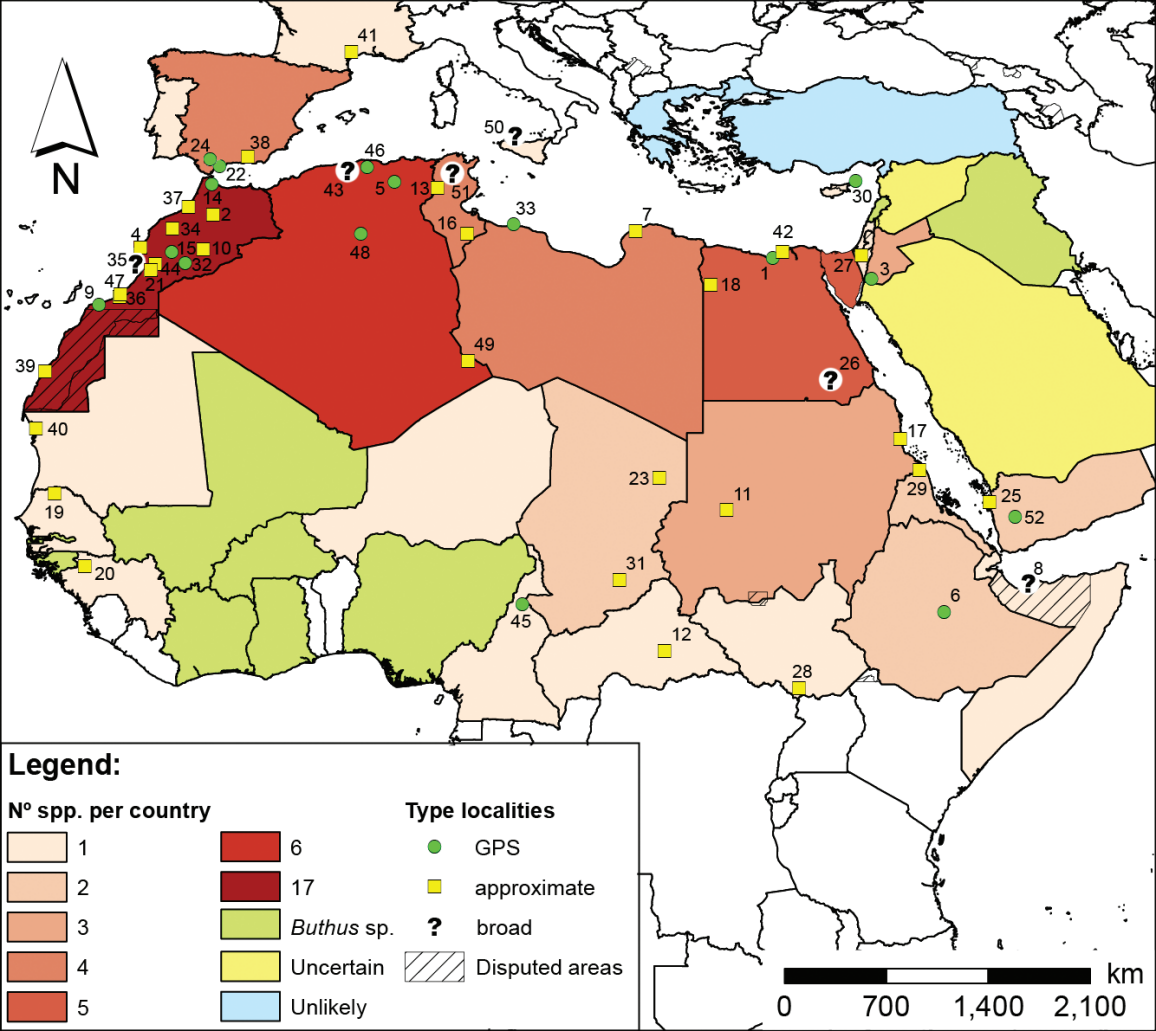
Map of *Buthus* species distribution, and the known number of species by country. Also depicted are the species’ type localities (numbers according to the species’ Catalogue and Table 2) where known or the best possible approximation. Actual distribution within each country can be much smaller, but detailed distribution information is unknown for the majority of species.

*Buthus* exhibits a wide distribution range, spanning over two biogeographic realms, the Palearctic (Western) and the Afrotropical (Udvardy 1975, Olson et al. 2001). Interestingly, none of the chorotypes proposed by Vigna Taglianti et al. (1999) satisfactorily describes *Buthus* distribution. The genus extends from the temperate Mediterranean areas of south-western Europe to the tropical and sub-tropical grasslands south of the Sahel and into the Horn of Africa, including the semi-arid and arid regions of North Africa and the Middle East (Fig. 2). Although first considered of European origin (Vachon 1952a), current data support the hypothesis that the centre of origin of the genus is North Africa (Lourenço 2002). North Africa harbours a disproportionate number of species (Fig. 2) as well as four of the five main genetic clades found in *Buthus*, as defined by mitochondrial DNA sequence variation (Sousa et al. 2012, Pedroso et al. 2013) (Fig. 7).

*Buthus* species are known from 17 countries in Africa: Algeria, Cameroon, Central African Republic, Chad, Egypt, Eritrea, Ethiopia, Guinea, Libya, Mauritania, Morocco, Niger, Senegal, Somalia, South Sudan, Sudan, and Tunisia; five countries in Asia: Cyprus, Egypt (Sinai), Israel, Jordan, and Yemen; and four European countries: France, Italy (Sicily), Portugal, and Spain (Fig. 2). Unidentified *Buthus* species have also been reported from Burkina Faso, Djibouti, Gambia, Ghana, Guinea-Bissau, Ivory Coast, Nigeria, Iraq, and Lebanon (Fig. 2). No records exist for Saudi Arabia or Syria, however the first might have been confused in the past with citations for the Arabian Peninsula (e.g. Vachon 1952a), although the existence of *Buthus* in either of these countries cannot be excluded. The frequently cited occurrence of *Buthus* in Iraq is based on a single specimen, deposited in the Czech National Museum of Natural History (Táborský 1934, Kovařík 1992). As such the actual distribution of the genus remains poorly delimited. Old records from mainland Greece and Turkey are highly doubtful, as these are well-studied areas with no recent *Buthus* collections (Ersen Yağmur pers. comm. for Turkey) (Fig. 2). The former records most likely refer to the genus *Mesobuthus* Vachon, 1949. As for the record for Malta, it was considered dubious by Fet and Lowe (2000), although other *Buthus* reported on islands that were previously regarded as doubtful have turned out to be correct, namely *B. kunti* Yağmur, Koc & Lourenço, 2011, from Cyprus, described from freshly collected material and *B. trinacrius* Lourenço & Rossi, 2013, from Sicily, based on 130-year-old material. Other island records include *B. occitanus* in the Columbrete islands (Castilla and Pons 2007) and *B. tunetanus* (Herbst, 1800) in the Tunisian islands of Djerba, Kerkena, and Zembra (Vachon 1952a).

*Buthus* is the type genus of the Buthidae C. L. Koch, 1837 (Koch 1837, 1850), the most diverse family within Scorpiones, with almost half of all known extant scorpion species (1101 of the 2311 known species) (Rein 2016). The Buthidae also includes most of the species venomous to humans (Chippaux and Goyffon 2008). The genus *Buthus* is the second oldest valid genus of the order Scorpiones C. L. Koch, 1837, only surpassed by the single genus created by Linnaeus in 1758, *Scorpio*, to accommodate all the scorpion species he described.

**Figure 3.**
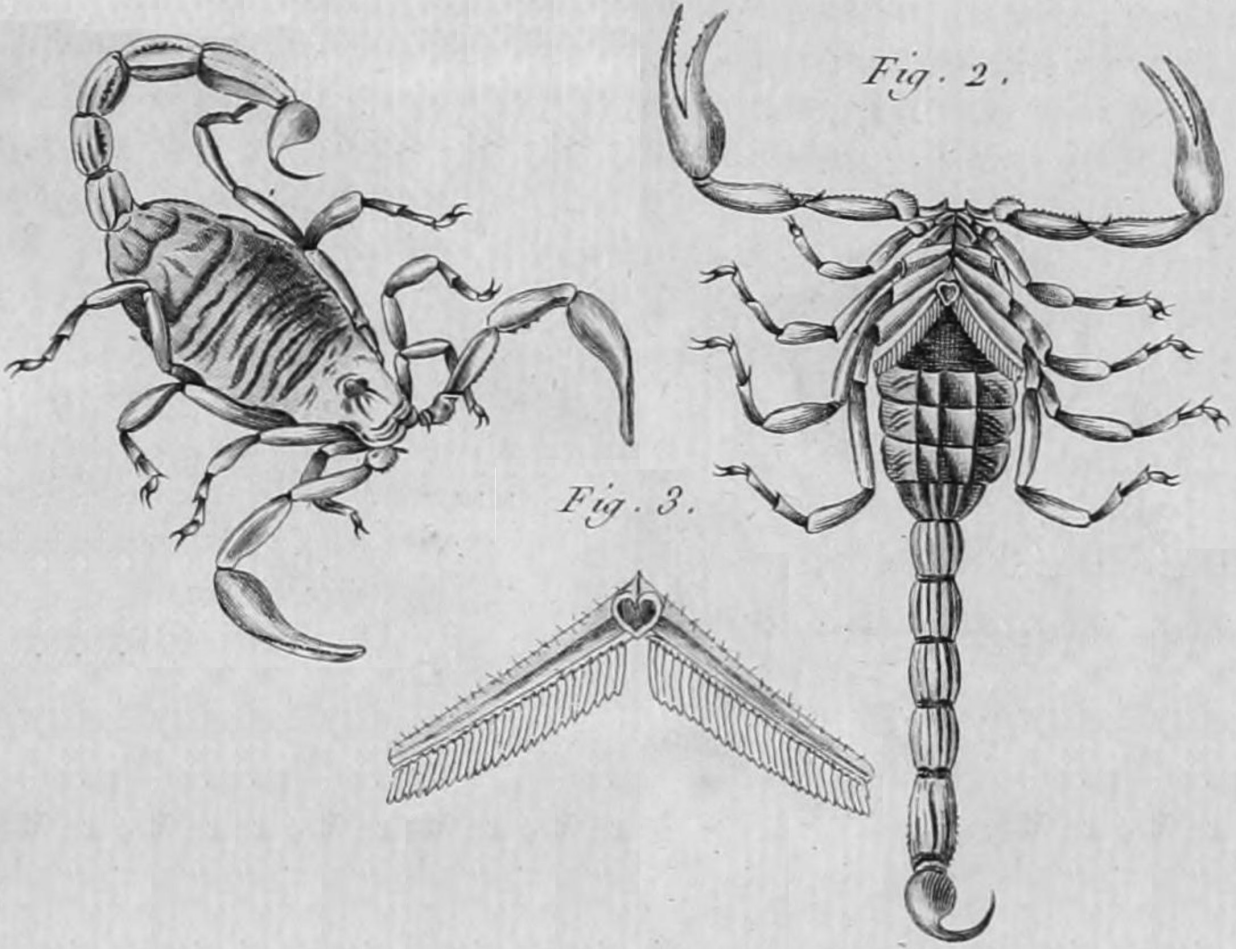
Original illustration of *Scorpio occitanus* (Amoreux 1789a). The work was retrieved from the Biodiversity Heritage Library and images were rearranged for compactness without re-scaling.

*Buthus* was first proposed by Leach (1815), with *Scorpio occitanus* Amoreux, 1789 as its type species (Fig. 3). The only diagnostic character for the genus proposed by Leach was the presence of eight eyes “*Oculi octo*.” (Leach 1815, page 391), apparently referring to the pair of central eyes and three pairs of lateral eyes. This character was mentioned as diagnostic for different scorpions early during Scorpiones taxonomy (De Geer 1778, Fabricius 1781). The same diagnostic character was used by Ehrenberg (in Hemprich and Ehrenberg 1828, 1829) for defining several genera and subgenera of scorpions with a varying number of eyes, ranging from six to 12. A more detailed explanation on the usage of the number of eyes in the classification of scorpions is given in Thorell (1876).

The poor description of Leach (1815) led to a rapid increase in the number of species included in the genus, which lacked any internal coherence. This taxonomic conundrum arose through the misidentification of the number of lateral eyes of *B. occitanus* (Amoreux, 1789), originally stated by Amoreux as three pairs. Several taxonomists of that century realised that there were actually four pairs of lateral eyes (e.g. Gervais 1844b; Simon 1879), but this information was not appreciated by some later authors. It has recently been shown that most Buthidae species (including *Buthus*) have five pairs of lateral eyes, although in many species two pairs of lateral eyes are much smaller in size and require extreme care and the help of UV light to be recognised (Yang et al. 2013; Loria and Prendini 2014). Ehrenberg (Hemprich and Ehrenberg 1828) modified the original meaning of the genus to include the species that are now part of *Heterometrus* Ehrenberg in Hemprich and Ehrenberg 1828 (Family Scorpionidae Latreille, 1802), all with five pairs of lateral eyes. Ehrenberg described several other genera that were soon synonymized with *Buthus*, at least by some taxonomists, which further exacerbated the taxonomic confusion within *Buthus*.

Because of the poor definition of the genus, many members (≈100 species) of the Buthidae family with no close relationship to the type species, were included in the genus *Buthus* up to the mid-20^th^ century (Vachon 1952a, Levy and Amitai 1980, Lourenço 2002). Unfortunately, this obsolete taxonomy is still in use, for example in many toxicology and venom related papers on scorpions (*e.g.* Gopalakrishnakone et al. 2015). From 1948 to 1951, Vachon conducted a major taxonomic revision of the genus (compiled in Vachon 1952a), providing a more informative and exclusive definition, retaining only the species that were morphologically similar to the type species and hence restricting also the distribution range of the genus. He proposed two main morphological characters that in combination separate *Buthus* from all other known Buthidae genera: the central-lateral and posterior-median prosomal keels fused in a lyra shape (character shared with *Cicileiurus* Teruel, 2007, *Leiurus* Ehrenberg in Hemprich and Ehrenberg 1828, *Mesobuthus* Vachon, 1950, and *Odontobuthus* Vachon, 1950, Fig. 4A), and the presence of only three granules on the tip of the movable finger (or tarsus) of the pedipalp chela (character shared with *Androctonus* Ehrenberg in Hemprich and Ehrenberg 1828, Fig. 4B).

**Figure 4.**
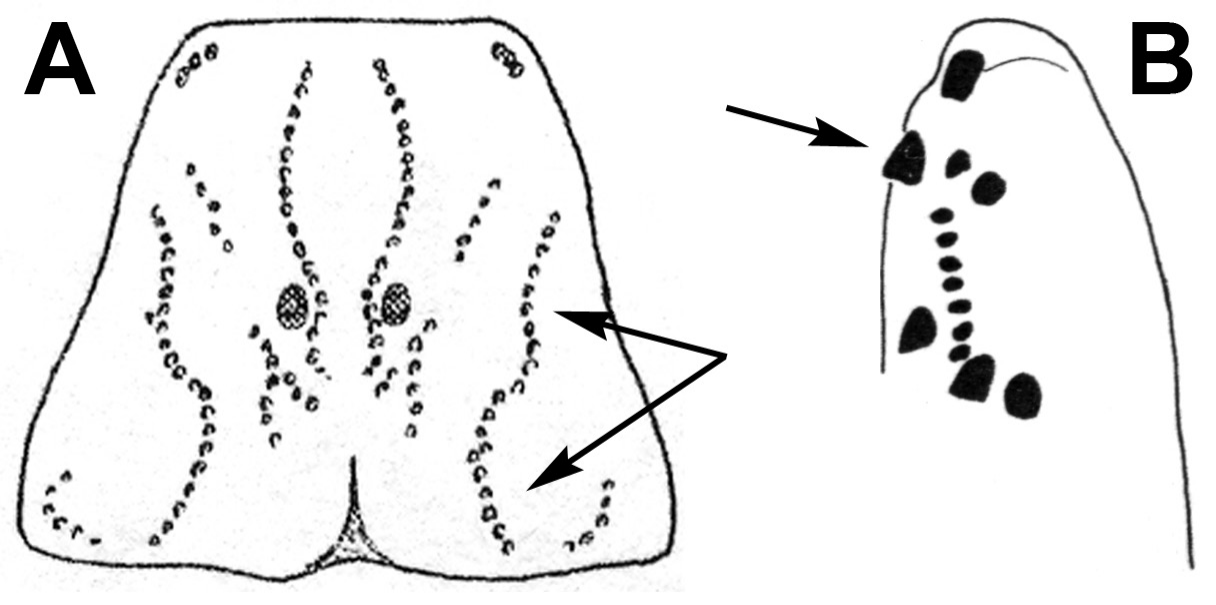
The two key morphological diagnostic characters of the genus *Buthus*. A- Prosoma carapace with lyra-shaped keels (Hjelle 1990); B - Tip of pedipalp movable finger highlighting the three distal granules (distal denticle not included) (Lourenço 2002).

While studying specimens from northwest Africa, Vachon recognized that the genus included a large amount of undescribed diversity. Vachon took a very conservative approach to *Buthus* taxonomy, recognizing only four species, further split in 12 subspecies, ten of which under *B. occitanus*, and naming four different varieties, along with other forms with no formal rank, all within *B. occitanus*. This was partly justified by Vachon’s view that *Buthus* species exhibited a large morphological plasticity, at least in the characters he used to diagnose the different taxa (Vachon 1952a). Stahnke (1972), in his key to Buthidae genera, recognized 21 species and subspecies in *Buthus*, without further explanation, although this is probably an error resulting from an outdated interpretation of the genus taxonomy. In accordance with the ICZN article 45, none of Vachon’s infra- subspecific varieties were included in the Catalogue of the Scorpions of the World (Fet et al. 2000). The *Buthus* Catalogue recognized as good five species and 12 subspecies, although the authors recognized that some taxa were probably not taxonomically good (Fet and Lowe 2000) Subsequently, Rossi (2015) transferred *Buthus insolitus* Borelli, 1925 to the recently erected genus *Gint* Kovařík et al., 2013. Lourenço (2003) marked a renewed interest in the taxonomy and diversity of the genus, describing six new species, some of which corresponding to Vachon’s infra-subspecific varieties.

**Figure 5.**
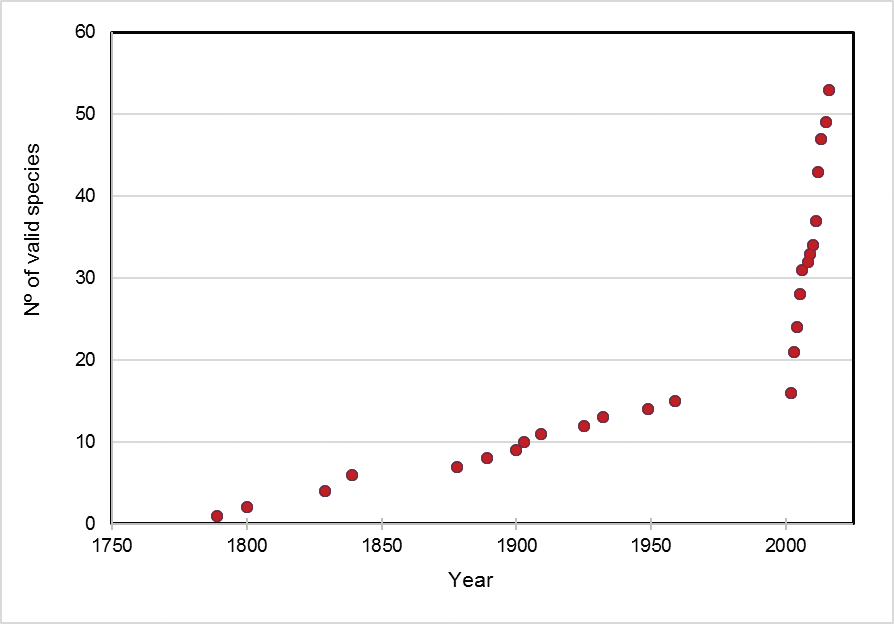
Cumulative number of valid named *Buthus* species. Only current valid species' names were plotted; in the year they were first described.

During the last 15 years, the rate of description of new *Buthus* species has increased exponentially (Fig. 5). At present, the genus is composed of 52 species, three of which were described in 2016, making it the fourth most diverse genus of Buthidae, only surpassed by the megadiverse scorpion genera *Tityus* C. L. Koch, 1836, *Centruroides* Marx, 1890 and *Ananteris* Thorell, 1891 (Rein 2016). Thirty authors have been involved in the description of recent *Buthus* species, and most species (21) have been described in collaborative studies. Wilson Lourenço is by far the most prolific author, having authored or co-authored 29 *Buthus* species, 55% of the total.

## Materials and methods

Nomenclature and measurements follow Stahnke (1970), except for trichobothriotaxy (Vachon 1974, Fet et al. 2005). All diagnostic morphological characters mentioned in the text refer to adults (or large sub adults) of both sexes, unless otherwise noted.

Most references prior to 1998 cited by Fet and Lowe (2000) are confirmed, but not all original literature could be obtained, and we made some corrections following comparisons with additional sources (Vachon 1952a, Lamoral 1979, Polis 1990, Hendrixson 2006, Dupré 2013). The criteria applied by Fet and Lowe (2000) citing both taxonomic and faunistic works are broadly followed. To the best of our knowledge we cited all works that follow these criteria up to November 2016. Fet and Lowe (2000) cited approximately 180 articles pertaining to the genus *Buthus*, we added approximately 80 new articles, ten of which were published before 1998.

Whenever possible, coordinates for the type localities are provided, using information available in articles or, if not available, finding approximate coordinates with the help of Google Maps (maps.google.com) and the GEOnet Names Server (geonames.nga.mil/gns/html). All coordinates are in WGS 1984 datum, in Latitude/Longitude format, in decimal degrees.

Collections abbreviation codes are listed below. Abbreviation codes follow Sabaj (2016), except for those marked with an asterisk that are not presented there.

ARPC* Andrea Rossi Private Collection, Massa, Italy

CBGP* Centre de Biologie pour la Gestion des Populations (UMR INRA, Cirad, IRD, Montpellier SupAgro), Montferrier-sur-Lez France, France

FKPC* František Kovařík Private Collection, Prague, Czech Republic

**MCSNB** Museo Civico di Scienze Naturali “Enrico Caffi”, Bergamo, Italy (formerly MSNB)

**CRBA** Centre de Recursos de Biodiversitat Animal of the Universitat de Barcelona, Barcelona, Spain

**MCVR** Museo Civico di Storia Naturale di Verona, Verona, Italy

**MCZ** Museum of Comparative Zoology, Harvard University, Cambridge, U.S.A.

**MHNG** Muséum d'histoire naturelle de Genève, Geneva, Switzerland

**MNCN** Museo Nacional de Ciencias Naturales, Madrid, Spain

**MNHN** Muséum national d’Histoire naturelle, Paris, France

**MRSN** Museo Regionale di Scienze Naturali di Torino, Turin, Italy

MTAS * Museum of the Turkish Arachnology Society, Ankara, Turkey

**MZUF** Museo di Storia naturale dell’Università di Firenze, sezione di Zoologia “La Specola”, Florence, Italy

**NHMUK** Natural History Museum, London; England, UK (formerly BMNH, British Museum of Natural History)

UCAM * Université Cadi Ayyad, Faculte des Sciences Semlalia, “Laboratoire Ecologie et Environnement”, Marrakech, Morocco (formerly Universite Cadi Ayyad, Faculte des Sciences, Semlalia, Depart. BioI., Lab. Ecol. Anim. Terrestre, Marrakech, Marocco)

UGA * University of Ghardaïa, Ghardaïa, Algeria

**ZIN** Zoological Institute, Russian Academy of Sciences, St. Petersburg, Russia (formerly ZISP)

**ZMB** Museum für Naturkunde - Leibniz-Institut für Evolutions und Biodiversitätsforschung, Berlin, Germany (formerly ZMBH)

**ZMH** Biozentrum Grindel und Zoologisches Museum, Hamburg, Germany

Additional abbreviations used in the text:

**a.s.l.** above sea level

**ICZN** International Code of Zoological Nomenclature

**IOS** incorrect original spelling

**ISS** incorrect subsequent spelling

**juv.** juvenile or juveniles

**M** male

**F** female

**MIS** misidentification

### The type species of the genus *Buthus*

**Figure 6.**
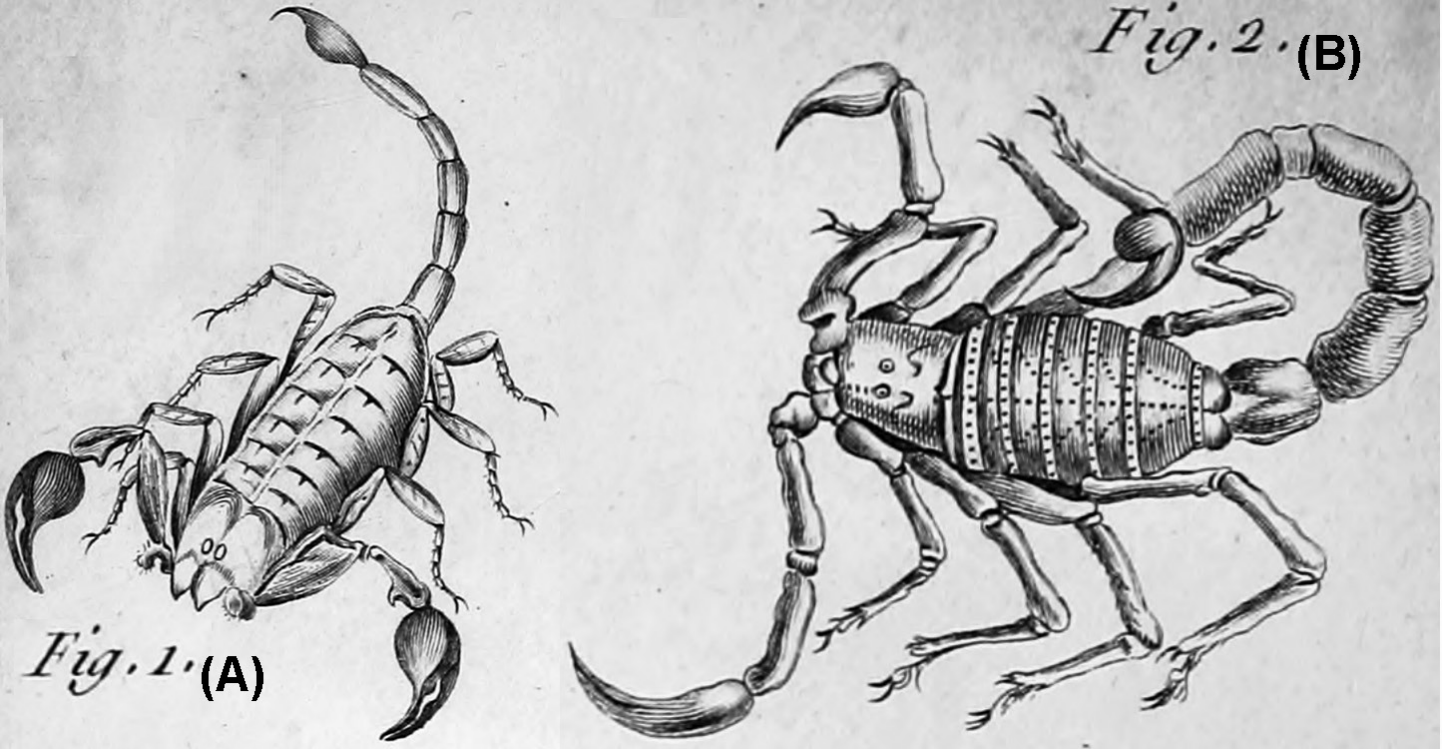
Original drawings of the habits of *Scorpio europaeus* Linnaeus, 1758 (A) and *S. occitanus* (B), according to Amoreux (1789b) reproduced from plate I of that work. These images are unfortunately rarely cited, as they are very informative regarding the reasoning of Amoreux while describing the new species.

It is worth mentioning here the taxonomic confusion that surrounded the first *Buthus* species. Leach first named the genus with *Scorpio occitanus* Amoreux, 1789 as the type species (Figs 3; 6B). Amoreux (Amoreux 1789b) described this species to accommodate a scorpion from Souvignargues, Occitanie, France. Amoreux also called the same species *Scorpio rufus*, although he, as the first reviewer, chose the name *S. occitanus* to be the correct name for the newly described species (Amoreux 1789a). Amoreux also referred to this species as Malpertius’ scorpion and, in his second paper where he gave a full description of the species, included drawings from this author (Maupertuis 1731) (Fig. 6B). It is clear from observing Fig. 6A that Amoreux was well aware of the differences between *Scorpio occitanus* and *S. europaeus* Linnaeus, 1758, which he considered a member of the genus *Euscorpius* Thorell, 1876. Unfortunately, the name *S. europaeus* Linnaeus, 1758 was subsequently used to refer to three different taxa: (1) *S. maculatus* De Geer, 1778 (now part of the genus *Isometrus* Ehrenberg, 1828) (Lönnberg 1898), (2) *S. occitanus* Amoreux, 1789 (Thorell 1876b), and (3) a *Euscorpius* species (Fet & Sissom, 2000). This taxonomical confusion was solved by ICZN decision 60 (ICZN 1957), article 1b that suppressed the name *europaeus*, Linnaeus, 1758 when used in combination with *Scorpio*, and article 4, that placed *Scorpio europaeus* Linnaeus, 1758 on the Official Index of Rejected and Invalid Specific Names in Zoology with the number 381. As such, the first author to use the name *Buthus europaeus* was Thorell in 1876, now a junior synonym of *B. occitanus* (Amoreux, 1789) (for further details see Braunwalder 1997, Fet et al. 2002).

Finally, although *Buthus* is considered the nominal genus of Buthidae, Koch (1837) used for the typification of the family, the species *Buthus spinnifer* Ehrenberg, 1828, which is currently the type species of the genus *Heterometrus* Ehrenberg, 1828 (Scorpionidae Latreille, 1802), and as such according to ICZN Article 65.2.1. [“type genus was misidentified (that is, interpreted in a sense other than that defined by its type species)] when the family-group name was established”) we will submit to the ICZN a petition to fix the type species of the Buthidae. This was not done by Fet et al. (2000) as the authors probably interpret it as ICZN Article 65.2.3. (“type genus was, when established, based on a type species then misidentified”), which does not necessarily require a ruling by the Commission.

### *Buthus* taxonomy

The revised classification of Sharma et al. (2015) is followed, based on the first phylogenomics study on extant scorpions, which resolved most relationships between scorpion families.

Class Arachnida Lamarck, 1801

Order Scorpiones C. L. Koch, 1850

Suborder Neoscorpiones Thorell & Lindström, 1885

Infraorder Orthosterni Pocock, 1911

Parvoder Buthida Soleglad & Fet, 2003

Superfamily Buthoidea C. L. Koch, 1837

Family Buthidae C. L. Koch, 1837

There are no subfamilies in use within the Buthidae, although many have been proposed and rejected (Fet et al. 2000, 2005). Fet et al. (2005) defined six groups within the Buthidae, and placed *Buthus* in the *Buthus* group along with 38 additional genera. The phylogenomic study of Sharma et al. (2015) provided strong support for most groups (although generic level sampling was limited), including the sister group relationship of the *Buthus* group with the remaining members of the familiy.

Vachon (1952a) considered *Androctonus* to be the sister taxa to *Buthus*. However, the only molecular phylogenetic study addressing the relationships between Buthidae genera that includes both genera (Fet et al. 2003), recovered *Buthus* as the sister taxa to a clade formed by *Androctonus* and *Leiurus*, albeit with low support.

There are no taxonomically distinct groups within the genus *Buthus*, although two “species complexes” are generally recognised. Vachon (1952a, p. 251) suggested that “*la «lignée» atlantis se sépare avec facilité de l’ensemble des autres Buthus par la forme élancée des appendices de la queue, de la vésicule et divers autres caractères que nos tableaux de détermination préciseront.*” This distinction was retained by Lourenço (2002, 2003) who referred to *B. occitanus* as a “«*complexe de forms*»”, and subsequently also adopted it in most subsequent taxonomic works describing new *Buthus* species. According to Lourenço and Geniez (2005), the two complexes are distinguished by the level of keel development, weaker in the *atlantis* group. However, Lourenço (2005a) subsequently wrote that *B. occitanus* from Europe has a weak keel development in contradiction with the previous morphological definition. This statement has been used by subsequent authors. For example, Rossi (2012) described *B. elongatus* Rossi, 2012 as belonging to the *occitanus* complex, but if the author had applied Vachon’s definition it should have included it in the *atlantis* complex because of the slender metasoma (at least its fifth segment) when compared to *B. occitanus*. None of the published molecular phylogenies of *Buthus* supports the existence of the *atlantis* complex. The results of Gantenbein and Largiadèr (2003) grouped *B. atlantis* within the species included in the *occitanus* complex. Although Lourenço and Vachon (2004) acknowledged the work of Gantenbein and Largiadèr (2003), they rejected the phylogenetic position of *B. atlantis* presented in this study.

Recently, based on the information provided by a *cox1* mtDNA tree, Sousa et al. (2012) and Pedroso et al. (2013) have defined a series of groups, (see Table 1). These groups have been renamed to facilitate communication and have been expanded to include all available molecular data with reliable species identifications.

**Table 1.** Current composition of the groups proposed by Sousa et al. (2012) and Pedroso et al. (2013) based on *cox1*. To date, only 19 out of the 52 valid named *Buthus* species (37%) have been analysed. *, assignment based on Sousa (2017).

Group	Species	Group	Species	Group	Species
***boumalenii***	*B. boumalenii*	***occitanus***	*B. atlantis*	***rochati***	*B. bonito*
			*B. elongatus*		*B. draa*
***mardochei***	*B. elmoutaouakili*		*B. ibericus*		*B. mariefranceae*
	*B. lienhardi*		*B. malhommei*		B. rochati*
	*B. mardochei*		*B. maroccanus*	***tunetanus***	B. chambiensis*
	*B. parroti*		*B. montanus*		B. pusillus*
			*B. occitanus*		*B. tunetatus*

**Figure 7.**
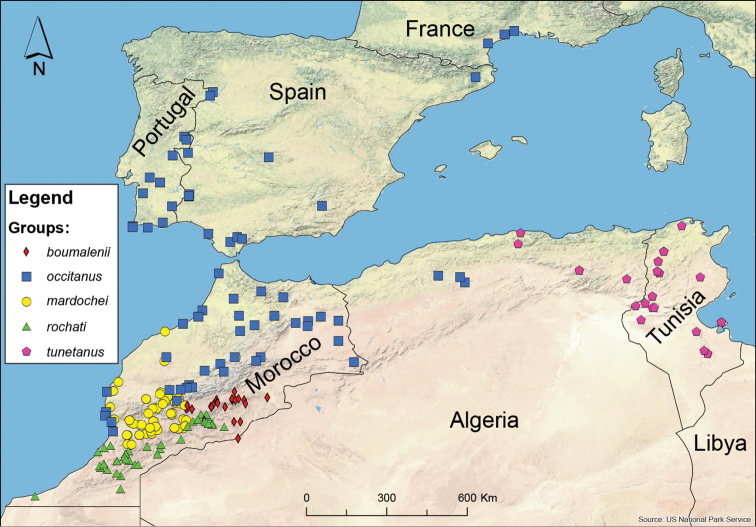
Map representing the five phylogenetic *Buthus cox1* groups in the Maghreb. Groups as defined by Sousa et al. (2012) and Pedroso et al. (2013), also including cox1 sequences from Gantenbein and Largiadèr (2003), Habel et al. (2012) and Husemann et al. (2012) (redrawing of Figure 1 from Pedroso et al. 2013).

In Fig. 7 the current distribution of these five groups in the Maghreb, the Iberian Peninsula, and southern France is presented, based exclusively on specimens with available molecular data. The group assignment does not necessarily correspond to the species assignment in the original publications.

### Diagnostic characters used in *Buthus* taxonomy

Several morphological traits have been used by recent authors as diagnostic characters (in the sense of Winston 1999) for *Buthus* species.

Colour is of limited utility in *Buthus* taxonomy, as the underlying colour varies in tones of yellow, orange, reddish or light brown within and between species. Only one species has a fully dark body, *Buthus maroccanus* Birula, 1903, in some cases even black. Other species also have the mesosoma of a darker colour than the rest of the body. Of greater taxonomic use are colour patterns, such as darker marks, over a lighter background colour, that can be present on the carapace, the mesosoma or the metasoma; the latter being the more informative.

**Figure 8.**
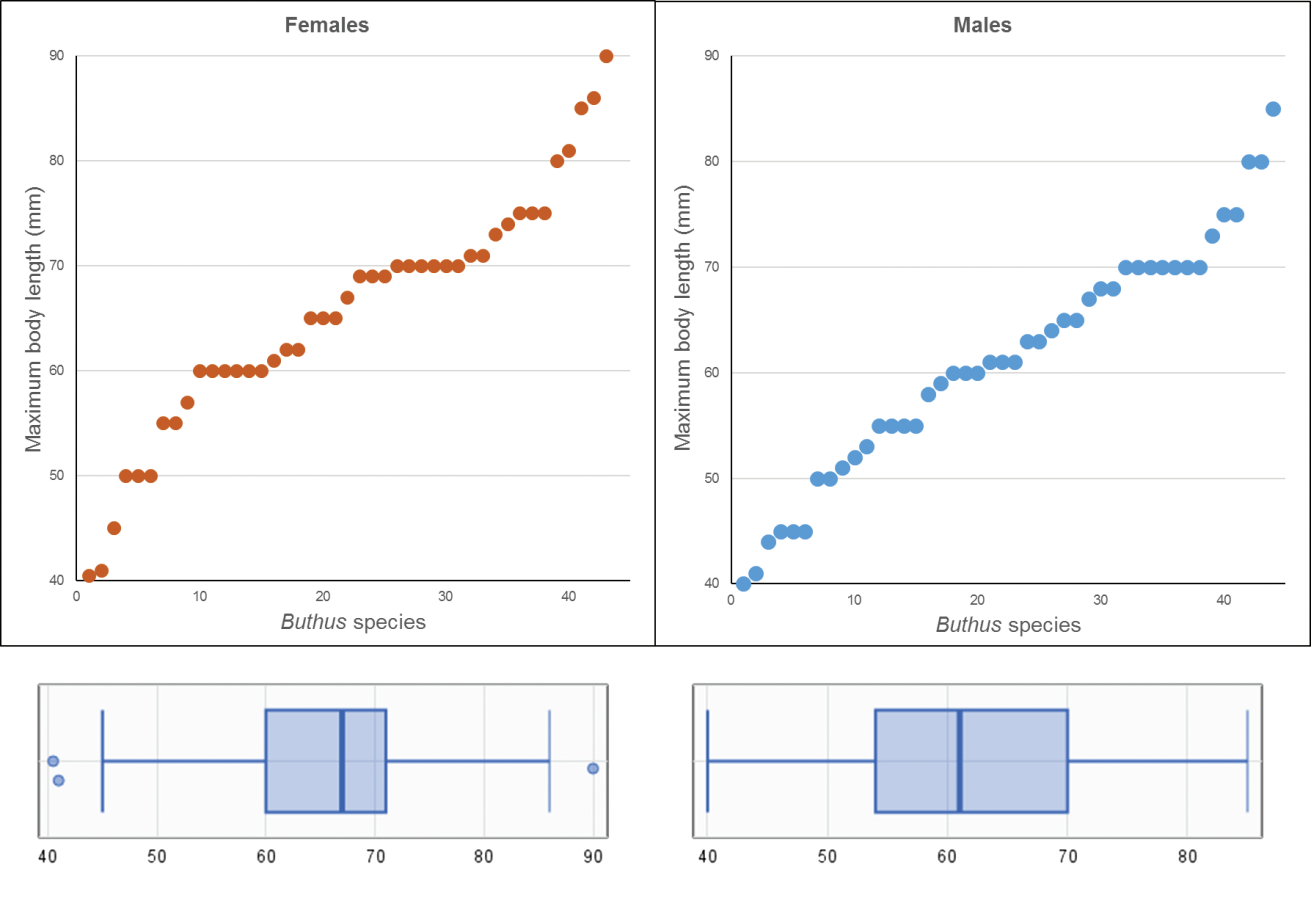
Chart and boxplot summary of *Buthus* species maximum sizes. Only the known maximum size per species is represented. Size information is only available for males from 44 species and females from 43 species. Some individual data might correspond to subadult specimens since this information is not always explicit in species descriptions.

Adult size may also be diagnostic (Fig. 8). *Buthus* adult body sizes range from 38 to 90 mm (telson included) (the maximum size of 110 mm reported by Vachon (1952a) is presumably a mistake). Most species have a maximum size between 60 and 70 mm in females, and 55 to 70 mm for males (Fig. 8). On the 5% percentiles we have the smaller species of *Buthus* (less than 45 mm long), and the larger species of *Buthus* (more than 85 mm for females and 80 mm long for males).

**Figure 9.**
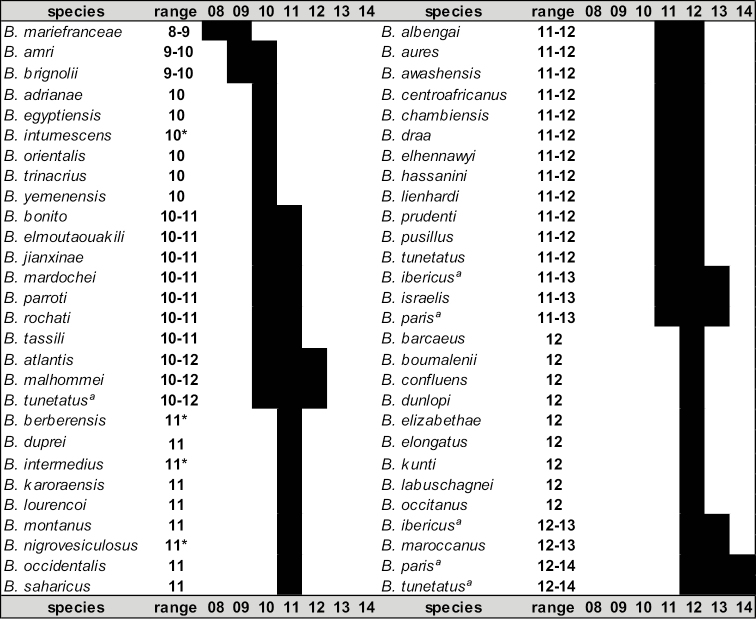
Graphical representation of the variation in the number of rows in the movable finger of all *Buthus* species. ª Species for which the bibliographic ranges are conflicting. * Number of rows in species identified by us from images of the type specimens may be underestimated.

**Figure 10.**
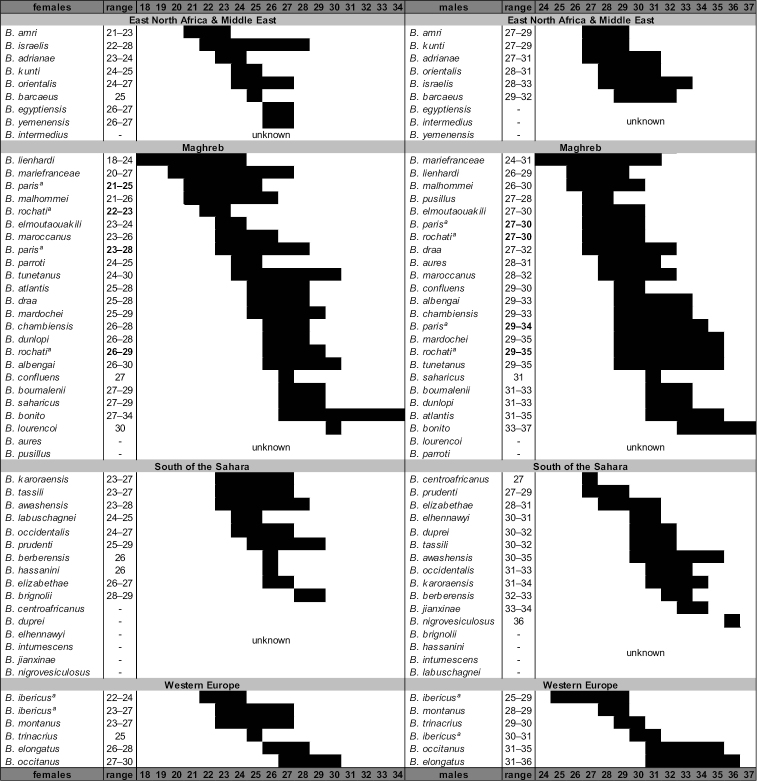
Graphical representation of the variation in pectinal teeth number of *Buthus* species arranged by geographical areas to facilitate comparison. All known Buthus species are represented, although female and male are ordered independently, from smaller to largest. ª Species for which the bibliographic ranges are conflicting.

Two additional meristic traits have been used as diagnostic characters, namely the number of rows of granules on the cutting edge of the movable finger of the pedipalp chela, and the number of pectinal teeth, a sexual dimorphic trait. Variation in the number of **rows of granules** is not very informative because species show an incremental overlap in the numbers of rows, which range from 8 to 14 (Fig. 9). **Pectinal teeth** number, although carrying a potentially greater amount of information as they have a wider range to vary from, is actually of limited usefulness because of the interspecific overlap (Fig. 10); female counts range from 18–34 and males from 24–37. Pectinal teeth number is also of limited use due to the lack of any information for several species and the unknown range of variability for many other *Buthus* species (Fig. 10). It should be noted, however, that *Buthus elizabethae* Lourenço, 2005 is unique in having male pectines that do not overlap in their proximal portion (Lourenço 2005a).

Trichobothria number and position are not useful for *Buthus* species diagnosis, as their location shows little variation and have as much intraspecific as interspecific variability (P. Sousa pers. obs.). Conversely, body **chaetotaxy** (other than trichobothria) is very useful for taxonomy. Vachon (1952a) defined three, albeit diffuse, states in *Buthus* body chaetotaxy: low (“*oligotriche*”), high (“*polytriche*”) and medium (“*mésotriche*”), and used the number of setae on the fifth segment of the metasoma as example of the ranges: low has three or fewer setae, high more than 5–6 setae and medium four setae, although this latter category was fluid. Confusion can further arise from the fact that these categories apply to the metasoma and the pedipalp, and in the same species these two body parts can have different ranges of chaetotaxy. Nevertheless, this is a useful trait, and one that needs to be explicitly stated in species descriptions to avoid misinterpretations. The chaetotaxy of the leg tarsi and mesosoma terguites is also useful.

Most other diagnostic traits in use for *Buthus* species are found in the metasoma and the pedipalp chela.

The length/width ratio of the **first metasomal** segment, which is typically square in most species but can be elongated or sturdy in certain species, is informative. This ratio is also applied to the **fifth metasomal** segment, and Vachon (1952a) further compared the ratios of the first and second segment, and sometimes even the third segment. The number of **keel rows** in the metasoma segments is also useful, with special attention paid to the presence, and in some cases the relative length (Vachon 1952a), of the median lateral keel in the second, third and fourth segments. Both the degree of development of the inferior median keels of the five segments (except perhaps the forth), and the existence of larger granules may also be used for taxonomic purposes. The number of lateral lobes in the **anal arch**, either two or three (the latter only in *B. atlantis* Pocock, 1889 and *B. lourencoi* Rossi, Tropea & Yagmur, 2013) may be misleading because in some species, or even specimens (Vachon 1952a), a third smaller lobe may be present between the two larger lobes, which has been interpreted as a third state (e.g. Sadine et al. 2015). For instance, Lourenço and Qi (2006) state that in *Buthus mariefranceae* Lourenço, 2003 the anal arch may sometimes have 3 lobes but this is mentioned neither in the original description nor in Vachon's descriptions. The relationship between the length of the aculeus and the length of the vesicle that form the **Telson** are also used in *Buthus* taxonomy. In most species, the aculeus is shorter than the vesicle, or as long as the vesicle at most. For a few species the aculeus is clearly shorter than the vesicle and for another handful of species, the aculeus is clearly longer. The states are defined here using a 10% difference threshold, but other authors have used a 5% difference. This ratio is correlated with the shape of the aculeus, also in use, which can be more or less curved.

**Figure 11.**
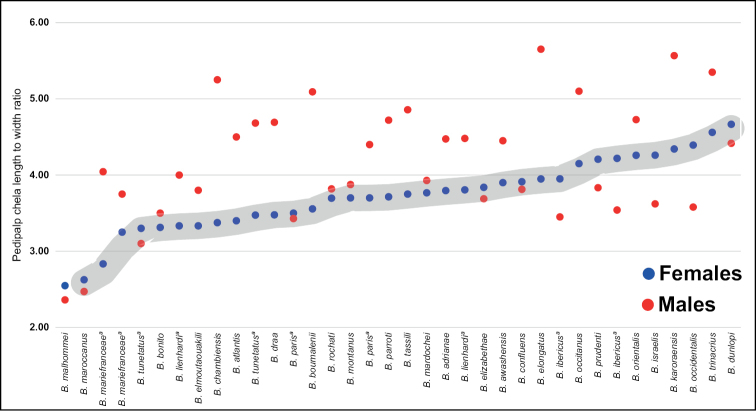
Graphical representation of the relation between female and male pedipalp chela aspect ratio in *Buthus* species. Only those species with available data from both sexes were plotted. The grey area represents species without perceived sexual dimorphism. Species with males above that area have slender pedipalp chela than females, while species with males plotted below have more robust pedipalp chela than females.

The shape of the **pedipalp chelae** in *Buthus* taxonomy has gained increased usage in recent years. The shape can be approximated by using the length to width ratio of the chela, which reflects its specific robustness or slenderness. However, in many *Buthus* species the chela shape is sexually dimorphic, a trait that was first used in a species key by Kovařík (2006), although its use goes back at least to Vachon (1952a). As a measure of sexual dimorphism the pedipalp chelae has three possible states: 1) no sexual dimorphism (male = female); 2) slender chela in male (male > female); 3) chela of male more robust (male < female). There is data available for 29 species (56% of the known species), and from these we can estimate that 38% do not have sexual dimorphism (+/- 10% threshold as a cut-off point), 52% of species have males with slender chelae and only 3 species (10%) show males with more robust chelae (Fig. 11). Interestingly, although chelae play a role in mating, defense, and as a sensory organ (van der Meijden et al. 2012), their prime importance in prey capture and handling (Polis 1990) may limit the slendering of female chelae, as these may be more prone to breakage (van der Meijden et al. 2012). For three of the 39 species, the available chelae data was contradictory, and they are further discussed below. Another useful pedipalp trait is the interrupted dorso-median keel of the patella in *Buthus rochati* Lourenço, 2003 (Vachon 1952a).

Several partial keys have been published over the years to assist *Buthus* species identification. However, due to the high rate of new species description (Fig. 5), they have become incomplete and even outdated in their taxonomy (Birula 1903, Vachon 1952a, Lourenço 2003, Lourenço and Vachon 2004, Kovařík 2006, Rossi 2012, Rossi et al. 2013, Teruel and Melic 2015). There are keys available for Morocco and the Maghreb by Birula (1903, only four species), Vachon (1952a, ten species in the Moroccan *Buthus* key), Lourenço (2003, 10+1 species), and Kovařík (2006, Tunisia, four species); for North Africa (excluding Morocco) by Rossi et al. (2013, 13 species), and for the Iberian Peninsula by Lourenço and Vachon (2004), Rossi (2012), and Teruel and Melic (2015).

Unfortunately, the identification of the majority of *Buthus* species remains difficult, in part because of the limited number of diagnostic characters and the incomplete knowledge regarding their intraspecific variation. Sexual dimorphism of pedipalp chelae is a promising trait, but for many species the male or female is still undescribed, which limits its applicability. Authors are urged to mention the variation on all the traits mentioned here (see Rossi et al. 2013 for a nice example) in future *Buthus* species descriptions.

Confirming the fast pace of new *Buthus* species descriptions, a new species, *Buthus danyii* Rossi, 2017 was published from Ghana (Rossi 2017) while this study was under revision. As such we were unable to include this species in the present update.

It is hoped that the present catalogue will facilitate a more precise, informative and comparative description of future species. *Buthus* are an important component of the scorpions’ fauna of North Africa and Western Europe, but it is only now becoming apparent that they are also diverse in the southern Sahara Desert, an area that should be prioritized in future surveys of *Buthus* scorpions.

## Catalogue

### Genus Buthus

Taxon classificationAnimaliaScorpionesButhidae

Leach, 1815

Buthus : Leach 1815: 391; Latreille 1817: 310; Gervais 1844b: 203; Peters 1861 (part): 513; Thorell 1876a (part): 82; Thorell 1876b: 7; Simon 1879: 95–96; Karsch 1886 (part): 77; Pocock 1890 (part): 122; Karsch 1891: 18; Kraepelin 1891 (part): 35–42; Pocock 1893 (part): 312; Kraepelin 1895 (part): 79–80; Laurie 1896b: 131; Lönnberg 1897b (part): 194; Kraepelin 1899 (part): 9; Pocock 1900a (part): 13; Simon 1910: 67–68; Birula 1917a (part): 20–24, 164; Birula 1917b (part): 55; Pavlovsky 1924 (part): 77; Kastner 1941 (part): 230; Vachon 1948a: 206–208; Vachon 1949a: 155–162; Vachon 1952a: 155, 241–246, fig. 579; Vachon 1963b: 164, fig. 10; Bücherl 1964: 57; Stahnke 1972: 132, fig. 20; Vachon 1974: 906; Levy and Amitai 1980: 14–15; Francke 1985: 6, 15; Sissom 1990: 101; Nenilin and Fet 1992: 17; Kovařík 1998: 106; Fet and Lowe 2000: 91; Lourenço 2016b: 3–4.

#### Type species

(by original designation). *Scorpio occitanus* Amoreux, 1789 [=*Buthus occitanus* (Amoreux, 1789)].

#### Etymology.

Leach did not provide an explanation for his selection of the genus name. A search on the original usage of the word may shed some light on the intended meaning. *Buthus* is the Latin form of the Greek name βοῦθος (*Bouthos*), an unusual name of a winning athlete of the ancient Pythian Games, mentioned by Hesychius and Aristotle (Müller 1848, Christesen 2007). The name was more familiar in antiquity when used in the adage “Βοῦθος περιφοιτᾷ”, translated to the Latin as “*Buthus obambulat*”, which translates into “*Buthus* who wanders”, which apparently was applied to stupid and simple people (Müller 1848, Christesen 2007). In Hofmann et al. (1698) the entry for *Buthus* reads “*athleta nobilis, qui bovem integrum unô die devorare solebat; unde natum proverbium in edaces, Buthus obambulat*” which roughly translates to “a noble athlete, who used to devour a great ox in a day, and who gave rise to the proverb, *Buthus obambulat*”. Noël (1824) entry for *Buthus* also refers to an athlete that devoured an ox in a single day, and that this voracity was the origin of the proverb “*Buthus obambulat*”, which according to the author refers to gluttony. Interestingly Noël also states that *Buthus*, in combination with “*βῦς, θúιεν* (*thuein*)”, also refers to sacrifice. This opinion shares roots with the meaning of two other words with similar etymology, *būthysĭa* (used by Nero) that translates to “sacrifice of an ox” and *būthytēs* (used by Pliny the Elder), that translates to a “sacrificed ox”, according to the Gaffiot Latin-French dictionary (Various 2016). Recently Dupré (2016) reached a similar conclusion, although he states that *Buthus* originates from the composition of the Greek word “Gr. *bous*, ox; - *thouéin* [Greek suffix?], killer”. Potentially therefore, *Buthus* refers to a stupid or voracious animal, an ox killer or to a sacrifice of an ox, from the latter two we can interpret it as a powerful and dangerous animal. In our opinion the later makes more sense and agrees well with what was known at the time about the potent venom of *Buthus* scorpions. As such, it is our opinion that *Buthus* is a singular masculine Latin word (of Greek origin), which Leach intended as homage to an ancient hero (a trend at that time), and that refers to an animal so venomous that it could kill an ox.

#### Distribution.

AFRICA: Algeria, Cameroon, Chad, Egypt, Eritrea, Ethiopia, Guinea, Ivory Coast, Libya, Mali, Mauritania, Morocco (including Western Sahara), Niger, Senegal, Somalia, South Sudan, Sudan, Tunisia. ?Guinea-Bissau, Nigeria, Burkina Faso, ?Gambia, Ghana, ?Djibouti. ASIA: Cyprus, Israel, Jordan, Yemen. ?Iraq, ?Lebanon, ?Saudi Arabia, ?Turkey. EUROPE: France, Italy (Sicily), Spain, Portugal. ?Malta, ?Greece (Corfu, Thessaly). All currently valid records of *Buthus* species per country are presented in Table 2. Figure 10 offers an additional zoom to the most diverse region of *Buthus* species diversity, the Maghreb.

**Table 2.** List of the countries for which there are valid records of the occurrence of *Buthus* species. The ID corresponds to the numbers used in Figures 1 and 10, and on the Catalogue. C.A.R. is the abbreviation of the Central African Republic.

ID	Taxa	France	Italy	Portugal	Spain	Algeria	Cameroon	C.A.R.	Chad	Djibouti	Egypt	Eritrea	Ethiopia	Guinea	Lybia	Mauritania	Morocco	Niger	Senegal	Somalia	S. Sudan	Sudan	Tunisia	Cyprus	Israel	Jordan	Yemen	Total (by taxa)
**01**	*B. adrianae*										**X**																	**1**
**02**	*B. albengai*																**X**											**1**
**03**	*B. amri*																									**X**		**1**
**04**	*B. atlantis*																**X**											**1**
**05**	*B. aures*					**X**																						**1**
**06**	*B. awashensis*												**X**															**1**
**07**	*B. barcaeus*														**X**													**1**
**08**	*B. berberensis*									**X**		**X**	**X**							**X**								**4**
**09**	*B. bonito*																**X**											**1**
**10**	*B. boumalenii*																**X**											**1**
**11**	*B. brignolii*																					**X**						**1**
**12**	*B. centroafricanus*							**X**																				**1**
**13**	*B. chambiensis*																						**X**					**1**
**14**	*B. confluens*																**X**											**1**
**15**	*B. draa*																**X**											**1**
**16**	*B. dunlopi*																						**X**					**1**
**17**	*B. duprei*																					**X**						**1**
**18**	*B. egyptiensis*										**X**																	**1**
**19**	*B. elhennawyi*																	**X**	**X**									**2**
**20**	*B. elizabethae*													**X**					**X**									**2**
**21**	*B. elmoutaouakili*																**1**											**1**
**22**	*B. elongatus*				**X**																							**1**
**23**	*B. hassanini*								**X**																			**1**
**24**	*B. ibericus*			**X**	**X**																							**2**
**25**	*B. intermedius*																										**X**	**1**
**26**	*B. intumescens*										**X**																	**1**
**27**	*B. israelis*										**X^1^**														**X**			**2**
**28**	*B. jianxinae*																				**X**							**1**
**29**	*B. karoraensis*											**X**																**1**
**30**	*B. kunti*																							**X**				**1**
**31**	*B. labuschagnei*								**X**																			**1**
**32**	*B. lienhardi*																**X**											**1**
**33**	*B. lourencoi*														**X**													**1**
**34**	*B. malhommei*																**X**											**1**
**35**	*B. mardochei*																**X**											**1**
**36**	*B. mariefranceae*																**X**											**1**
**37**	*B. maroccanus*																**X**											**1**
**38**	*B. montanus*				**X**																							**1**
**39**	*B. nigrovesiculosus*																**X^2^**											**1**
**40**	*B. occidentalis*															**X**												**1**
**41**	*B. occitanus*	**X**			**X**																							**2**
**42**	*B. orientalis*										**X**																	**1**
**43**	*B. paris*					**X**											**X**						**X**					**3**
**44**	*B. parroti*																**X**											**1**
**45**	*B. prudenti*						**X**																					**1**
**46**	*B. pusillus*					**X**																						**1**
**47**	*B. rochati*																**X**											**1**
**48**	*B. saharicus*					**X**																						**1**
**49**	*B. tassili*					**X**									**X**													**2**
**50**	*B. trinacrius*		**X^3^**																									**1**
**51**	*B. tunetatus*					**X**									**X**		**X**						**X**					**4**
**52**	*B. yemenensis*																										**X**	**1**
**Total (by country)**		**1**	**1**	**1**	**4**	**6**	**1**	**1**	**2**	**1**	**5**	**1**	**2**	**1**	**4**	**1**	**17**	**1**	**2**	**1**	**1**	**2**	**4**	**1**	**1**	**1**	**2**	

^1^ Sinai; ^2^ Western Sahara; ^3^ Sicily

#### Remarks.

There are several old records of *Buthus*, marked with a question mark above, which have never been found again (independently of the material in which they were based being lost or not). As such, many have not been checked since the genus was reduced in scope by Vachon (1949), or those localities remain doubtful because no *Buthus* has been collected there since. This is of special significance in countries like Greece and Turkey that have been in recent years reasonably well prospected. Type specimens for several *Buthus* species described early on were not designated or have since become lost, but this does not necessarily represent a taxonomic problem. For example *B. occitanus* has no type specimen (Fet and Lowe 2000), but its type locality is well established and no other *Buthus* species occurs nearby. In this case the designation of a neotype is not justified under the ICZN (article 75.2). However, this is not the case for other species that have neither type specimens nor localities, and that we will further discuss below.

### 1. Buthus adrianae

Taxon classificationAnimaliaScorpionesButhidae

Rossi, 2013

Buthus adrianae : Rossi 2013: 188–191, fig. 1–2; Rossi, Tropea and Yağmur 2013: 3; 5, 8.

#### Type material.

1 adult M holotype (MCSNB N° 14011), El-Hamam (30.8300°, 29.3150°), Alexandria, Egypt. Paratypes: 1 adult M and 1 adult F (ARPC), same locality.

#### Distribution.

known only from the type locality.

### 2. Buthus albengai

Taxon classificationAnimaliaScorpionesButhidae

Lourenço, 2003

Buthus albengai : Lourenço 2003: 902–904, fig. 70–74; Lourenço and Geniez 2005: 5; Aboumaâd et al. 2014: 6; Touloun et al. 2014: 76; Lourenço 2016b: fig. 3.

#### Type material.

1 F holotype (MHNG), Ito Plateau (approx. 33.51°, -5.3°), Ifrane, Morocco. Paratypes: 3 F, same locality; 2 juv., Ifrane (Cedars woods); 1 M, 1 F juv., region north of Kenifra (all in MHNG).

#### Distribution.

known from an area in Morocco that extends from ifrane to Kenifra.

#### Remarks.

It is one of the largest known *Buthus* species. Records by Habel et al. (2012) south of the High-Atlas are most likely misidentifications.

**Figure 12.**
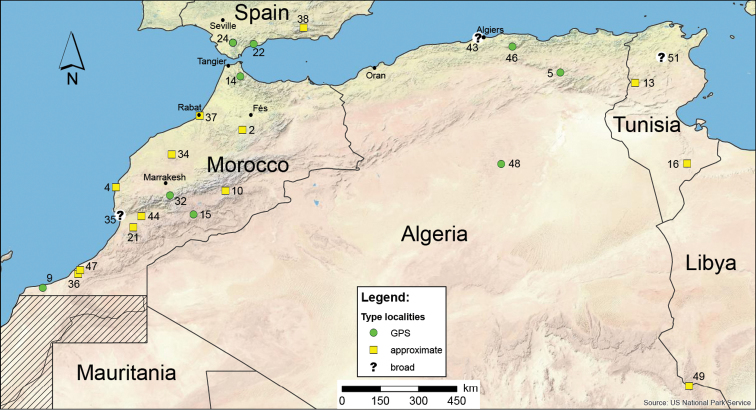
Map of North Africa Maghrebian *Buthus* species’ type localities (numbers according to the Catalogue and Table 2) whenever known or the best possible approximation at present.

### 3. Buthus amri

Taxon classificationAnimaliaScorpionesButhidae

Lourenço, Yağmur & Duhem, 2010

Buthus amri : Lourenço, Yağmur and Duhem 2010: 96–99, fig. 1–5; Lourenço 2013: 65; Lourenço and Rossi 2013: 9; Amr 2015: 186.

#### Type material.

1 M holotype (MNHN), Wadi Rum Desert (29.5363°, 35.4136°), Aqaba, Jordan. Paratypes: 1 adult F (MNHN), 2 adult F, 1 subadult F, 2 subadult M, 3 juv. (MTAS), all from the same locality.

#### Distribution.

known only from the type locality.

### 4. Buthus atlantis

Taxon classificationAnimaliaScorpionesButhidae

Pocock, 1889

= Tityus tenuimanusBanks 1910: 189 (synonymized by Lourenço and Francke 1984: 428). 1 F holotype (MCZ), Buena Vista Lake, California, USA (incorrect locality).
Buthus atlantis : Pocock 1889b: 340–341, pl. XV, fig. 4; Birula 1896: 244; Kraepelin 1891: 197, 199; Birula 1903: 107–108; Werner 1932: 300–305; Vachon 1949a: 162–169,fig. 345, 347, 349, 351, 354, 355, 357–362; Vachon 1952a: 254–255, fig. 345, 347, 349, 351, 354, 355, 357–362; Malhomme 1954: 25; Bücherl 1964: 57; Pérez 1974: 22; Levy and Amitai 1980: 15; EIHennawy 1992: 98, 119; Kovařík 1998: 106; Fet and Lowe 2000: 91; Lourenço 2005a: 233–234; Lourenço and Geniez 2005: 5; Stockmann and Ythier 2010: 360–361; Stockmann 2015: fig. 5.
Buthus Atlantis (sic): Aboumaâd et al. 2014: 5.
Buthus atlantis atlantis : Vachon 1949a: 166–168; Vachon 1952a: 252, 254; Le Corroller 1967: 63; Kovařík 1995: 20; Fet and Lowe 2000: 91; Lourenço 2003: 883–885, fig. 18–22; Touloun et al. 2001: 2; Gantenbein and Largiadèr 2003: 120, 122; Touloun 2012: 43, fig. 9A.
Buthus occitanus atlantis : Kraepelin 1899: 26–27; Werner 1934b: 86–87, fig. 5; Schenkel 1949: 186–187.
Buthus (Buthus) atlantis : Birula 1910: 145; Birula 1917a: 213, 223.
Tityus tenuimanus : Cox 1921: 12; Ewing 1928: 22; Mello-Leitão 1931: 121, 140; MelloLeitão 1939: 60, 64, 71; Comstock 1940: 27; Mello-Leitão 1945: 308; Gertsch and Soleglad 1966: 2; Hjelle 1972: 28; Lourenço and Francke 1984: 427, fig. 10–12.

#### Type material.

1 F holotype (NHMUK), Essaouira (formerly Mogador) (approx. 31.49°, -9.76°), Morocco.

#### Distribution.

known to occur only in sandy dune habitats close to the Atlantic Ocean in Morocco, between Essaouira and Agadir.

#### Remarks.

It is the largest known *Buthus* species.

### 5. Buthus aures

Taxon classificationAnimaliaScorpionesButhidae

Lourenço & Sadine, 2016

Buthus aures Lourenço and Sadine 2016: 14–17, fig. 4–13.

#### Type material.

1 M holotype (MNHN), Batna region (35.5319°, 5.9194°), Aurès Mountains, Algeria. 1 M paratype (UGA), same locality.

#### Distribution.

known only from the type locality.

### 6. Buthus awashensis

Taxon classificationAnimaliaScorpionesButhidae

Kovařík, 2011

Buthus awashensis : Kovařík 2011: 1–3, 5–8, fig. 5–16.
Buthus occitanus (MIS): Kovařík and Whitman 2005 (part): 106.

#### Type material.

1 M holotype (FKCP), Metahara (approx. 8.900°, 39.900°), Oromia, Ethiopia. Paratypes: 34 M, 34 F, 36 juv. (FKCP), all from the same locality; 1 M (FKCP), Dire Dawa, Ethiopia.

#### Distribution.

know only from two Ethiopian localities, more than 200 km apart.

#### Remarks.

The pedipalp chela length-to-width ratio given by the author for the type material suggest that some animals exhibit sexual dimorphism while others do not. If this is true, the utility of this ratio as a diagnostic character in *Buthus* would be compromised. Alternatively, it may be due simply to the use of immature specimens.

### 7. Buthus barcaeus

Taxon classificationAnimaliaScorpionesButhidae

Birula, 1909

Buthus occitanus barcaeus : Birula 1909: 508–511. fig. A, C; Borelli 1914a: 155–156; Borelli 1924: 5–7; Borelli 1928: 351; Caporiacco 1932: 395; Borelli 1934: 169; Caporiacco 1937: 345; Pérez 1974: 23; Levy and Amitai 1980: 16; El-Hennawy 1992: 98, 120; Kovařík 1998: 106; Fet and Lowe 2000: 95; Kovařík 2002: 5.
Buthus (Buthus) occitanus barcaeus : Birula 1910: 156; Birula 1917a: 223.
Buthus barcaeus : Kovařík 2006: 3, fig. 6; Kaltsas et al. 2008: 215; Lourenço and Cloudsley-Thompson 2012: 15; Lourenço and Simon 2012: 11; Rossi, Tropea and Yağmur 2013: 3–5, 7.

#### Type material.

4 M, 1 F juv., syntypes (ZIN), Barca (approx. 32.48°, 20.83°), 5 km E from Benghazi (Cyrenaica), Libya.

#### Distribution.

know from several localities along the Mediterranean coast of Libya.

#### Remarks.

The specimens present in the MNHN (F n° 4896), captured in Barca and identified by Vachon in 1974, have no intermediary keel on the fourth metasomal segment, which casts doubts about the use of the character in the diagnosis of *B. barcaeus*.

### 8. Buthus berberensis

Taxon classificationAnimaliaScorpionesButhidae

Pocock, 1900

= Buthus occitanus zeylensisPocock 1900b: 56–57 (synonymized by Levy and Amitai 1980: 16). 1 F holotype (NHMUK), Zeyla (northwestern Somaliland), Somalia.
Buthus occitanus berberensis : Birula 1903: 106–107; Birula 1909: 510; Birula 1910: 118; Kraepelin 1903: 558; Borelli 1904:·2–3; Giltay 1929: 196; Moriggi 1941: 84; Lamoral and Reynders 1975: 505; Levy and Amitai 1980: 16; EI-Hennawy: 1992: 98, 120; Kovařík 1998: 106; Fet and Lowe 2000: 95; Kovařík 2003 (part): 138.
Buthus occitanus zeylensis : Kraepelin 1903: 558–559; Borelli 1919: 363; Borelli 1931: 218; Caporiacco 1936: 137; Moriggi 1941: 84; Lamoral and Reynders 1975: 505–506; EI-Hennawy 1992: 98, 121–122; Kovařík 1998: 106.
Buthus berberensis : Lourenço 2008: 46; Kovařík 2011: 4–6.
Buthus (Buthus) occitanus berberensis : Birula 1917a: 123.

#### Type material.

1 M holotype (NHMUK), Somaliland, Somalia.

#### Distribution.

know from Djibouti, Eritrea, Ethiopia and Somalia, although Birula (1903) recorded toponyms that are old and difficult to map.

#### Remarks.

Lourenço (2008) stated that *B. o. zeylensis* might be a distinct species from *B. berberensis*, but that further material was required to confirm this possibility. Kovařík (2011) considered *B. o. zeylensis* a colour morph of *B. berberensis* present in juveniles and some males.

### 9. Buthus bonito

Taxon classificationAnimaliaScorpionesButhidae

Lourenço & Geniez, 2005

**Figure 13.**
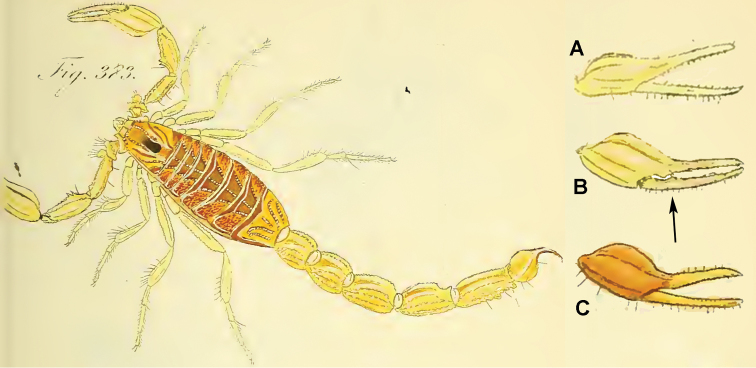
Reproduction of C. L. Koch’s 1839 *B. halius* figure 382 (plate CLXIII). Right pedipalp che-lae detail from three *Buthus* species published in the same work: **A** *B. paris* (as *Androctonus clytoneus*), fig. 384 (same plate) **B** *B. halius* with an arrow pinpointing the basal lobe **C** *B. paris*, fig. 352 (pl. CLI). All images were taken as provided by the pdf copy available in the BHL, which was made available by the Ernst Mayr Library of the Museum of Comparative Zoology, Harvard University.

= Buthus sabulicolaTouloun 2012: 46, 48–58, fig.10, 13, 14 (**Syn. n.**). 1 F holotype (MNHN), Khnifiss lagoon, Tan-Tan Province, Morocco.
Buthus bonito : Lourenço and Geniez 2005: 1–5, fig. 1–8, 10; Touloun et al. 2008: 3–4, fig.1; Stockmann and Ythier 2010: 362–363; Pedroso et al. 2013: 300; Aboumaâd et al. 2014: 6; Touloun et al. 2016: 880, fig. 2D.

#### Type material.

1 M holotype (MNHN N° RS8669), Khnifiss lagoon (approx. 27.93°, -12.34°), Tarfaya, Morocco. Paratypes: 2 F (MNHN N° RS8670), from the same locality.

#### Distribution.

known from the Atlantic coast of Morocco south of Tan-Tan extending almost to Dakhla in the Western Sahara (Touloun et al. 2016).

#### Remarks.

Although the type material of *B. sabulicola* was collected in 2002 by Touloun, Stockmann and Slimani, the species was not formally described until the publication of the PhD thesis of Oulaid Touloun in 2012. The type specimens of *B. bonito* and *B. sabulicola* are from the exact same locality, the Khnifiss lagoon, and both descriptions are almost identical. Touloun et al. (2016), probably by mistake, indicated that the fifth metasomal segment and telson are darkened in *B. bonito*, but the trait does not appear in the accompanying figure.

### 10. Buthus boumalenii

Taxon classificationAnimaliaScorpionesButhidae

Touloun & Boumezzough, 2011

https://science.mnhn.fr/institution/mnhn/collection/rs/item/rs8891

Buthus boumalenii : Touloun and Boumezzough 2011a: 183–186, fig. 2–7; Pedroso et al. 2013: 300; Aboumaâd et al. 2014: 6; El Hidan et al. 2016: 4.

#### Type material.

1 F holotype (UCAM), Tineghir (approx. 31.366°, -5.905°), Boumalene, Morocco. Paratypes: 1 M (UCAM), 1 M, 1 F (MNHN, N° RS8891), all from the same locality.

#### Distribution.

known only from the Boumalne region of Morocco (El Hidan et al. 2016).

#### Remarks.

This species is the only known representative of a phylogenetic lineage present east of the High Atlas Mountains of Morocco. Because of its phylogenetic uniqueness, the conservation of this species should have top priority.

### 11. Buthus brignolii

Taxon classificationAnimaliaScorpionesButhidae

Lourenço, 2003

Buthus brignolii : Lourenço 2003: 905–907, fig. 75–79; Rossi and Tropea 2016a: 4.

#### Type material.

1 F holotype (MHNG), Djebel Meidob (approx. 15.21°, 26.44°), Darfur, Sudan.

#### Distribution.

known only from the type locality.

#### Remarks.

it is one of the four known “inland island” species of *Buthus* that have been found in the Mountainous regions in the heart of the Sahara Desert.

### 12. Buthus centroafricanus

Taxon classificationAnimaliaScorpionesButhidae

Lourenço, 2016

https://science.mnhn.fr/institution/mnhn/collection/rs/item/rs9069

Buthus centroafricanus : Lourenço 2016a: 73–77, fig. 1–11.

#### Type material.

1 M holotype (MNHN), Between Bria and Yalinga (as Jalinga) (approx. 6.52°, 22.62°), Province Haute-Kotto, Central African Republic.

#### Distribution.

known only from the type locality.

### 13. Buthus chambiensis

Taxon classificationAnimaliaScorpionesButhidae

Kovařík, 2006

Buthus chambiensis : Kovařík 2006: 1–3, fig. 2–5; Rossi, Tropea and Yağmur 2013: 3, 7.

#### Type material.

1 M holotype (FKCP), Djebel Chambi Mountain (approx. 35.17°, 8.56°), Kasserine Province, Tunisia. Paratypes: 1 M juv., 2 F, 1 juv., all from the same locality.

#### Distribution.

known only from the type locality.

### 14. Buthus confluens

Taxon classificationAnimaliaScorpionesButhidae

Lourenço, Touloun & Boumezzough, 2012

https://science.mnhn.fr/institution/mnhn/collection/rs/item/rs8919

https://science.mnhn.fr/institution/mnhn/collection/rs/item/rs8920

Buthus confluens : Lourenço, Touloun and Boumezzough 2012: 22–24, fig. 1–11; Touloun et al. 2014: 76–77.

#### Type material.

1 M holotype (MNHN N° RS8919), Alhamra (35.39529°, -05.37181°), Tétouan, Morocco. Paratypes: 1 F (MNHN N° RS8920), 1 M (UCAM), all from the same locality.

#### Distribution.

known from several localities in the Tingitana Pensinsula of Morocco, but also further to the south.

#### Remarks.

Based on the colour pattern and pigmentation, the original authors suggested that *B. confluens* was the closest phylogenetic relative in Morocco to *B. ibericus*, from the Iberian Peninsula. However, because the presence of three dark bands on the metasoma is shared among several *Buthus* species, this claim should be further confirmed with additional data.

### 15. Buthus draa

Taxon classificationAnimaliaScorpionesButhidae

Lourenço & Slimani, 2004

https://science.mnhn.fr/institution/mnhn/collection/rs/item/rs8694

Buthus draa : Lourenço and Slimani 2004: 166–169, fig. 1–7; Lourenço, Sun and Zhu 2009: p. 72, fig. 2–6; Stockmann and Ythier 2010: 362–363; Touloun and Boumezzough 2011b: 186; Habel et al. 2012: 2, 4; Sousa et al. 2012: 68–69; Pedroso et al. 2013: 300; Yang et al. 2013: 2; Aboumaâd et al. 2014: 6; El Hidan et al. 2016: 4.
Buthus occitanus tunetanus neeli (MIS): Touloun et al. 1999: 1–2.
Buthus tassili (MIS): Touloun 2012: 37, 40–41, fig.7.

#### Type material.

1 M holotype (UCAM), Taznakht (30.51853°, -7.02595°), Ouarzazate, Morocco. Paratypes: 1 M, 2 F (UCAM), 1 M, 1 F (ZMH), 2 M, 1 F (MNHN), all from the same locality; 1 M (ZMH), Aït Bassou; 2 F juv. (ZMH), Aït Ounzar Oulad Aissa; 1 M (ZMH), near Agdez; 1 M (ZMH), Oulad Hlal. The ZMH accession number for all paratypes is A7/03.

#### Distribution.

*B. draa* can be found in the upper part of the Draa River, probably at elevations below 1500 m a.s.l.

#### Remarks.

*B. draa* shares with *B. tassili* and *B. nigrovesiculosus* the presence of a darkened fifth metasoma segment and telson.

### 16. Buthus dunlopi

Taxon classificationAnimaliaScorpionesButhidae

Kovařík, 2006

Buthus dunlopi : Kovařík 2006: 2–3, 6, fig. 7–8; Rossi, Tropea and Yağmur 2013: 5, 7.

#### Type material.

1 M holotype (FKCP), Remada (approx. 32.31°, 10.39°), Tataouine, Tunisia. Paratypes: 1 M, 3 F (FKCP), same locality.

#### Distribution.

known only from the type locality.

### 17. Buthus duprei

Taxon classificationAnimaliaScorpionesButhidae

Rossi & Tropea, 2016

http://zoobank.org/86EDFE2D-B287-4DCD-BD37-8B99FC58915C

Buthus duprei Rossi and Tropea 2016b: 25–28, fig. 1–12.

#### Type material.

1 M holotype (MCVR), Port Sudan (approx. 19.59°, 37.19°), Sudan. Paratype: 1 M juv. (ARPC N° 0809), same locality.

#### Distribution.

known only from the type locality.

### 18. Buthus egyptiensis

Taxon classificationAnimaliaScorpionesButhidae

Lourenço, 2012

Buthus egyptiensis : Lourenço and Cloudsley-Thompson 2012: 12–16, fig. 1–7; Lourenço and Simon 2012: 12; Rossi 2013: 191–192; Rossi, Tropea and Yağmur 2013: 4, 7.

#### Type material.

1 F holotype (ZMH N° A20/12), Siwa (approx. 29.17°, 25.46°), Egypt.

#### Distribution.

known only from the type locality.

#### Remarks.

One of the four known “inland island” species of *Buthus* that have been found within the Sahara Desert, although in this case from an Oasis. It is also one of the largest known *Buthus* species.

### 19. Buthus elhennawyi

Taxon classificationAnimaliaScorpionesButhidae

Lourenço, 2005

https://science.mnhn.fr/institution/mnhn/collection/rs/item/rs8637

Buthus elhennawyi : Lourenço 2005b: 246–249, fig. 1–7; Lourenço and Leguin 2012: 8.

#### Type material.

1 M holotype (ZMH N° A42/05), Fété-Olé (as Félé-Olé) (16.233°, -15.099°), Ferlo, Senegal. Paratype: 1 M (MNHN N° RS8637), Rosi (as Rossi), Niger.

#### Distribution.

this species is known from Niger and Senegal, from a single locality in each country, which are almost 2,000 Km apart.

#### Remarks.

We used the location of Fété-Olé given in Vincke et al. (2010), a locality that has been part of long term ecological studies, to map this locality, instead of the original spelling “Félé-Olé”.

### 20. Buthus elizabethae

Taxon classificationAnimaliaScorpionesButhidae

Lourenço, 2005

https://science.mnhn.fr/institution/mnhn/collection/rs/item/rs8638

Buthus elizabethae : Lourenço 2005a: 230–235, fig. 1–12, Lourenço 2005b: 249.

#### Type material.

1 M holotype (ZMH N° A36/05), S.W. of Gaoual (approx. 11.71°, -13.22°), Boké, Guinea. Paratypes: 1 F (ZMH N° A37/05), same locality; 1 M, 1 F (MNHN), Niokolo-Koba National Park, Senegal.

#### Distribution.

this species is known from Guinea and Senegal,

#### Remarks.

Given the geographical proximity, it is possible that the *Buthus* material reported to have been found in Guinea-Bissau might very well correspond to this species. Unfortunately the Guinea-Bissau material was lost in a fire, and hence only newly collected material could confirm this possibility.

### 21. Buthus elmoutaouakili

Taxon classificationAnimaliaScorpionesButhidae

Lourenço & Qi, 2006

Buthus elmoutaouakili : Lourenço and Qi 2006: 288–291, fig. 1–11; Habel et al. 2012 (part): 2, 3; Husemann et al. 2012 (part): 2, 4–5; Touloun and Boumezzough 2011b: 11–12, fig. 2C; Aboumaâd et al. 2014: 6.
Buthus occitanus mardochei alluaudi : Vachon 1949c: 363–367, fig. 409–416; Vachon 1952a: 291–295, fig. 409–416; Le Corroller 1967: 63; Pérez 1974: 23; Touloun 2012: 39, 57.

#### Type material.

1 M holotype (ZMH N° A18/06), Ait Baha (approx. 30.07°, -9.15°), Chtouka Aït Baha, Morocco.

#### Distribution.

this species seems to be widely distributed across the western portion of the Anti-Atlas, although some misidentifications with *Buthus parroti* cannot be excluded.

#### Remarks.

According to ICZN article 45.5, Vachon’s (1949) infrasubspecific name is unavailable. Although the name was published before 1961, it was only used as infrasubspecific by all subsequent authors.

### 22. Buthus elongatus

Taxon classificationAnimaliaScorpionesButhidae

Rossi, 2012

Buthus elongatus : Rossi 2012: 273–278, fig. 1–6; Teruel and Melic 2015: 5–9.

#### Type material.

1 adult M holotype (MZUF N° 1432), Sierra Blanca (36.533°, 4.900°), Marbella, Malaga Province, Spain. Paratypes: 1 adult F (ARPC), same locality; 1 M, 1 F (MZUF N° 875), Playa del Alicate (36.499°, 4.818), Marbella, Malaga Province, Spain.

#### Distribution.

this species is known from the southern Iberian Mediterranean coast, close to Marbella.

#### Remarks.

The second locality given by Rossi as Alicante (sic), had a typographic error, as the coordinates given by the author, together with their map in Fig. 7, provide sufficient evidence for the correct mapping of this locality. Both localities are under severe anthropomorphic pressure.

### 23. Buthus hassanini

Taxon classificationAnimaliaScorpionesButhidae

Lourenço, Duhem & Cloudsley-Thompson, 2012

https://science.mnhn.fr/institution/mnhn/collection/rs/item/rs8927

Buthus hassanini : Lourenço et al. 2012: 319–321, 323, fig. 35–42.

#### Type material.

1 F (MNHN N° RS8927), Biti Tehëc (approx. 17.187°, 22.288°), Ennedi Plateau, Chad.

#### Distribution.

known only from the type locality.

#### Remarks.

Another of the four known “inland island” species of *Buthus* that have been found in a Mountainous region in the heart of the Sahara Desert. The type locality was pinpointed following the map provided by the authors (fig. 90).

### 24. Buthus ibericus

Taxon classificationAnimaliaScorpionesButhidae

Lourenço & Vachon, 2004, nomen protectum

https://science.mnhn.fr/institution/mnhn/collection/rs/item/rs8605

= Buthus halius (C. L. Koch, 1839) **(*nomen oblitum*) (comb. n., syn. n.)**. Holotype lost according to Fet and Lowe (2000), Portugal.
Buthus ibericus Lourenço & Vachon, 2004: 88–91, fig. 31–42, Fernández 2004: 222; Teruel and Pérez-Bote 2005: 273–276, fig. 1; Armas and González-Moliné 2009: 553–554; Fet 2010: 4; Sousa et al. 2010: 207; Rossi 2012: 274–275, 277–278; Pedroso et al. 2013: 300; Teruel and Melic 2015: 6–9.
Androctonus halius C. L. Koch 1839a: 69–70, pl.CLXIII, fig. 383; Gervais 1844a: 43; C. L. Koch 1850: 90; Simon 1879: 96.
Buthus occitanus (MIS): Berejano and Pérez-Bote 2002: 59.

#### Type material.

1 M holotype (MNHN N° RS8605), San José del Valle (36.6247°, -5.6646°), Cádiz, Spain. Paratypes: 2 F (MNHN, N° RS8654), 1 M, 2 F (CRBA, N° CRBA-21826), and 2 F (MNCN N° 20.02/14857), all from the same locality.

#### Distribution.

this species seems to have a wide distribution range in the western part of the Iberian Peninsula, although the limits of its distribution remain poorly defined.

#### Remarks.

*B. ibericus* was first described from Spain and subsequently reported for Portugal (e.g. Sousa et al. 2010, Rossi 2012). Simon (1879) synonymized *Androctonus halius* with *B. occitanus* based on its type locality, which was wrongly stated to be Spain (page 98), because Simon considered *B. occitanus* (as *B. europaeus*) to be the only good species in Spain. Koch’s original description is not by itself enough to synonymize both species. However, Koch’s fig. 383 illustration of *Androctonus halius* includes a basal lobe in the movable finger (Fig. 13a and B), which is the key diagnostic character for *B. ibericus* (Lourenço and Vachon 2004, Rossi 2012), together with its type locality (Portugal), supports this synonymy. Nonetheless, according to the I.C.Z.N. article 23.9, the junior synonym can remain valid to maintain taxonomic stability. To our knowledge the name *B. halius* has not been used since 1879 (article 23.9.1.1), and more than 25 works have been published in the past 12 years by more than 10 authors using the name *B. ibericus* (article 23.9.1.2). Not all works are cited here because they are neither taxonomic nor faunistic. As such we propose to maintain as valid the junior synonym *B. ibericus* (*nomen protectum*) according to prevailing usage (article 23.9.1), and to consider the senior synonym *B. halius* a *nomen oblitum*. Rossi, 2012 (page 278), for *B. ibericus*, states erroneously “Sexual dimorphism is not noticeable in the chela manus”, which is in contrast to the original descriptions of both Koch (1839) and Lourenço and Vachon (2004) and to the complementary description in Teruel and Pérez-Bote (2005). In both works it can be observed that the male manus is more bulbous than the female’s, which results in the males having a smaller length to width ratio. The type locality of *B. ibericus* was precisely located following the indications of Iñigo Sánchez, the original collector.

### 25. Buthus intermedius

Taxon classificationAnimaliaScorpionesButhidae

(Ehrenberg in Hemprich and Ehrenberg 1829)

http://www.systax.org/en/details/spm/88132

Androctonus (Leirus) tunetanus intermedius : Ehrenberg in Hemprich and Ehrenberg 1829: 354; Braunwalder and Fet 1998: 33–34.
Androctonus (Leiurus) tunetanus intumescens (MIS): Kovařík 2006: 10.
Androctonus (Liurus) tunetanus intermedius : Ehrenberg in Hemprich and Ehrenberg 1831: (pages not numbered).
Androctonus occitanus intermedius : Gervais 1844a: 42.
Buthus (Buthus) occitanus intermedius : Birula 1917a: 228.
Buthus occitanus intermedius : Peréz 1974: 23.
Buthus intermedius (Ehrenberg): Lourenço 2008: 46–47.

#### Type material.

1 F (in bad conditions) (ZMB N° 146), Al Luhayyah (as Lohaie), Yemen.

#### Distribution.

Known only from the type locality.

#### Remarks.

Fet and Lowe (2000) considered the locality as probably wrong since at that time no other *Buthus* had been collected again in Yemen. However, Lourenço's (2008) *Buthus yemenensis* revalidated Ehrenberg species’ by providing concrete proof for the existence of *Buthus* species in this country. Doubst about *B. intermedius* type locality were the only evidence given by Kovařík (2006) for its synonimization with *Buthus intumescens* (Ehrenberg in Hemprich and Ehrenberg 1829).

### 26. Buthus intumescens

Taxon classificationAnimaliaScorpionesButhidae

(Ehrenberg in Hemprich and Ehrenberg 1829)

http://www.systax.org/en/details/spm/88133

Androctonus (Leiurus) tunetanus intumescens : Ehrenberg in Hemprich and Ehrenberg 1829: 354; Braunwalder and Fet 1998: 33.
Androctonus (Liurus) tunetanus intumescens : Ehrenberg in Hemprich and Ehrenberg 1831 (pages not numbered); Moritz and Fischer 1980: 316.
Androctonus occitanus intumescens : Gervais 1844a: 42.
Buthus intumescens : Kovařík 2006 (part): 10–11, 15, fig. 20; Kaltsas et al. 2008 (part): 215; Rossi 2013: 191–192; Rossi, Tropea and Yağmur 2013: 3, 6–8.

#### Type material.

1 (sex unknown) (in bad conditions) (ZMB N° 145), Egypt.

#### Remarks.

known only from a single specimen.

### 27. Buthus israelis

Taxon classificationAnimaliaScorpionesButhidae

Shulov & Amitai, 1959

Buthus occitanus mardochei israelis : Shulov and Amitai 1959: 219–225, fig. 1–3.
Buthus occitanus israelis : Pérez 1974: 23; Vachon and Kinzelbach 1987: 101; Fet and Lowe 2000: 95; Skutelsky 1995: 46; Skutelsky 1996: 50
Buthus occitanus israelis (Shulov & Amitai, 1959): Levy and Amitai 1980: 16–21, fig. 25–29; EI-Hennawy 1992: 101, 120; Kovařík 2006: 10; Lourenço, Yağmur and Duhem 2010: 96.
Buthus intumescens (MIS): Kovařík 2006 (part): 10–11.
Buthus israelis : Lourenço, Yağmur and Duhem 2010: 96–97; Yağmur, Koç and Lourenço 2011: 29.
Buthus israelis (Shulov & Amitai, 1959): Rossi 2013: 191–192; Rossi, Tropea and Yağmur 2013: 2–3, 6–7.
Buthus occitanus mardochei israelis : Vachon 1966: 211; Kovařík 2006: 10; Lourenço, Yağmur and Duhem 2010: 95.
Buthus occitanus typicus : Bodenheimer 1937: 235.

#### Type material.

holotype lost (sex unknown), Mash’abbe Sade (as Mashavei Sadé) (approx. 31°, 34.78°), Negev desert, Israel.

#### Distribution.

This species has been recorded in Egypt (the Sinai Peninsula) and Israel. Notwithstanding, Kovařík (2006) considered this species to be a junior synonym of *B. intumescens*.

#### Remarks.

Levy and Amitai (1980) did not designate any neotype when they re-described the species. In contrast to the several infrasubspecific taxa described by Vachon that are unavailable according to the ICZN, *B. o. israelis* is an available name according to the ICZN article 45.6.4.1 (“a name that is infrasubspecific under Article 45.6.4 is nevertheless deemed to be subspecific from its original publication if, before 1985, it was either adopted as the valid name of a species or subspecies or was treated as a senior homonym”), which is the present case as Levy and Amitai redescribed this taxon before 1985, and thus articles 45.5 and 45.5.1 do not apply. Several authors wrongly report the original authors of the species in parenthesis. The use of parenthesis is only to be made when a species is changed from one genus to another (ICZN article 51.3), which is clearly not the case with *B. israelis*.

### 28. Buthus jianxinae

Taxon classificationAnimaliaScorpionesButhidae

Lourenço, 2005

https://science.mnhn.fr/institution/mnhn/collection/rs/item/rs8175

Buthus jianxinae : Lourenço 2005c: 22–23, fig. 1–12.

#### Type material.

1 M holotype (MNHN N° RS8175), Loka (approx. 4.21°, 30.91°), Equatoria, South Sudan.

#### Distribution.

known only from the type locality.

### 29. Buthus karoraensis

Taxon classificationAnimaliaScorpionesButhidae

Rossi & Tropea, 2016

http://zoobank.org/9EA2BC5A-9E0B-4457-8E32-EA3C3FAA0A74

Buthus karoraensis : Rossi and Tropea 2016a: 4–7, fig. 1–13; Rossi and Tropea 2016b: 25.
Buthus occitanus berberensis (MIS): Kovařík 2003 (part): 138.
Buthus occitanus (MIS): Kovařík and Whitman 2005 (part): 106.

#### Type material.

1 M holotype (MZUF N° 610), Karora (17.703°, 38.365°) (small enclave in the Eritrean-Sudan border), Eritrea. Paratypes: 1 M, 4 F (MZUF N° 610); 1 M (MCSNB: N° 12749), 1 F (MCSNB: N° 12748), all from the same locality.

#### Distribution.

known only from the type locality.

### 30. Buthus kunti

Taxon classificationAnimaliaScorpionesButhidae

Yağmur, Koç & Lourenço, 2011

http://zoobank.org/96DA8302-0891-4EF8-8D5B-DA8275325908

https://science.mnhn.fr/institution/mnhn/collection/rs/item/rs8892

Buthus kunti : Yağmur, Koç and Lourenço 2011: 29–33, fig. 1–12.
Buthus europaeus (MIS): Simon 1879: 97.
Buthus occitanus (MIS): Kraepelin 1891 (part): 199.
Buthus sp .: Levy and Amitai 1980: 21.

#### Type material.

1 F holotype (MTAS), Rizokarpaso (Dipkarpaz) (35.58472°, 34.42306°), Karpaz Region, Cyprus. Paratypes: 1 M juv. (MTAS), Zafer. 1 M juv. (MNHN N° RS8892), Güzelyurt.

#### Distribution.

the species is only known from the northern portion of Cyprus.

#### Remarks.

according to Yağmur, Koç and Lourenço (2011), this species is rare in the island.

### 31. Buthus labuschagnei

Taxon classificationAnimaliaScorpionesButhidae

Lourenço, 2015

https://science.mnhn.fr/institution/mnhn/collection/rs/item/rs8992

Buthus labuschagnei Lourenço 2015: 22–24, fig. 13–22.

#### Type material.

1 F holotype (MNHN N° RS8992), Zakouma (Zakouma National Park) (approx. 10.89°, 19.82°), Salamat Region, Chad.

#### Distribution.

known only from the type locality.

### 32. Buthus lienhardi

Taxon classificationAnimaliaScorpionesButhidae

Lourenço, 2003

Buthus lienhardi : Lourenço 2003: 899–902, fig. 62–69; Stockmann and Ythier 2010: 362–363; Touloun and Boumezzough 2011a: 186; Touloun 2012: 37, fig.5C; Aboumaâd et al. 2014: 6.
Buthus occitanus tunetatus Lepineyi : Vachon 1949: 353–359, fig. 393–400; Vachon 1952a: 281–286, fig. 393–400;
Buthus occitanus tunetatus lepineyi : Malhomme 1954: 29–30; Le Corroller 1967: 63; Peréz 1974: 22; Touloun et al. 1999: 1; Touloun et al. 2001: 2; Touloun 2012: 37.
Buthus occitanus tunetatus (MIS): Touloun 2012: 104, 108.

#### Type material.

1 M holotype (MHNG), Oukaimeden (approx. 31.201°, -7.861°), Marrakech, Morocco. Paratypes: 1 F, 2 juv. (MHNG), same locality.

#### Distribution.

the species is known from a wide range across the High-Atlas Mountains.

#### Remarks.

Vachon (1949) infrasubspecific name is not available as explained previously.

### 33. Buthus lourencoi

Taxon classificationAnimaliaScorpionesButhidae

Rossi, Tropea & Yağmur, 2013

http://zoobank.org/82B4235D-820E-4FE2-8AFC-6E0B4E28334D

Buthus lourencoi : Rossi et al. 2013: 2–3, fig. 3–10.
Buthus occitanus (MIS): Kovařík and Whitman 2005 (part): 106.

#### Type material.

1 adult F holotype (MZUF N° 783), Mellaha (approx. 32.896°, 13.285°), Tripoli, Libya

#### Distribution.

known only from the type locality.

#### Remarks.

The type locality is now part of the large city of Tripoli. It is fairly unlikely that the species still occur within the boundaries of the city given the level of urban development. However, Mellaha, which was originally a military airport, is now the Mitiga International Airport, where large patches of unconstructed ground that may be suitable fot the species still exist.

### 34. Buthus malhommei

Taxon classificationAnimaliaScorpionesButhidae

Vachon, 1949

Buthus occitanus malhommei Vachon 1949: 376; Vachon 1952a: 304–308, fig. 433–444; Fet and Lowe 2000: 95; Touloun et al. 2001: 2; Touloun 2012: 35, 104, 108, fig. 5A.
Buthus malhommei : Lourenço 2003: 887–889, fig. 33–38; Stockmann and Ythier 2010: 364–365 (MIS); Sousa et al. 2012: 68–69; Aboumaâd et al. 2014: 5.

#### Type material.

3 M, 3 F, 7 juv., syntypes (MNHN), Mechra ben Abbou (approx. 32.646°, -7.800°), Settat, Morocco.

#### Distribution.

Toulon (2012) greatly expanded the known distribution of this species along the basin of the Oum er Rbia River.

### 35. Buthus mardochei

Taxon classificationAnimaliaScorpionesButhidae

Simon, 1878

Buthus mardoche (IOS): Simon 1878: 159–160; Simon 1879: 100; Kraepelin 1891: 199; Birula, 1896: 244
Buthus (Buthus) mardoche (IOS): Birula 1910: 145–146; Birula 1917a: 223 (“dubious species”); Werner 1932: 300–305.
Buthus occitanus mardochei : Vachon 1949c (part): 358–363, fig. 400–408; Vachon 1952a (part): 286–295, fig. 401–408; Malhomme 1954: 28–29; Pérez 1974: 22; Levy and Amitai 1980: 16; El-Hennawy 1992: 98, 120; Kovařík 1995: 20; Gantenbein et al. 1998a: 51; Gantenbein et al. 1998b: 33–39; Kovařík 1998: 106; Fet and Lowe 2000: 96; Touloun et al. 2001: 2; Gantenbein and Largiadèr 2003 (part): 120, 122.
Buthus occitanus mardochei mardochei : Le Corroller, 1967. 63; Touloun 2012: 39, 104, 108, fig.5D.
Buthus mardochei : Lourenço 2003: 889, fig. 39; Stockmann and Ythier 2010: 364–365; Aboumaâd et al. 2014: 5.

#### Type material.

1 F (MNHN N° RS1771, damaged), southern Morocco.

#### Distribution.

this species appears to have a distribution parallel to that of *B. atlantis*, between Essaouira and Agadir, but is found further inland and away from the Atlantic coast.

#### Remarks.

Vachon (1949d: 358) corrected what he considered Simon's incorrect original spelling of “mardoche” to “mardochei”, since the form was named as a patronym after its collector, Rabbi Mardoché. Nevertheless, it is our understanding that this was an unjustified emendation, because the ICZN article 31.1 admit the use of a noun in apposition as was the case with “mardoche”, however the ICZN article 33.2.3.1 admits the prevalence of this emendation as it continues to be attributed to “the original author and date” and is “in prevailing usage” and as such we refrain from any change to the name. Vachon (1949, 1952) also established that the species occurs roughly between Essaouira and Agadir, but not near the coast where it is replaced by *B. atlantis*.

### 36. Buthus mariefranceae

Taxon classificationAnimaliaScorpionesButhidae

Lourenço, 2003

Buthus mariefranceae : Lourenço 2003: 889–893, fig. 40–46; Lourenço and Qi 2006: 291; Stockmann and Ythier 2010: 364–365; Sousa et al. 2012: 68–69; Touloun 2012: 40; Pedroso et al. 2013: 300; Aboumaâd et al. 2014: 6.
Buthus occitanus mardochei mimeuri : Vachon 1949c: 367–373, fig. 417–425; Vachon 1952a: 295–301, fig. 417–425; Le Corroller 1967: 63; Pérez 1974: 23; Touloun 2012: 40, 57.

#### Type material.

1 F holotype (MHNG), Tan-Tan (approx. 28.43°, -11.1°), Guelmim Region, Morocco. Paratypes: 5 M, 4 F, 2 F juv. (MHNG), Goulimine.

#### Distribution.

this species has a large distribution in Morocco, east and south of the Anti-Atlas Mountain.

#### Remarks.

Vachon (1949) infrasubspecific name is not available as explained above.

### 37. Buthus maroccanus

Taxon classificationAnimaliaScorpionesButhidae

Birula, 1903

= Prionurus tingitanus: Pallary 1928a: 350–351, fig. 4 (synonymized by Vachon 1949b: 281). Syntype, sex unknown (MNHN), Rabat, Morocco (Vachon, 1949, 1952).
Buthus occitanus maroccanus : Birula 1903: 106.
Buthus europaeus (MIS): Hirst 1925 (part): 416.
Buthus (Buthus) occitanus maroccanus : Birula 1910: 145; Birula 1917a: 223.
Buthus occitanus maroccanus : Giltay 1929: 196; Werner 1929: 31–32.
Buthus maroccanus : Werner 1932: 299; Werner 1934b: 84; Vachon 1949b: 281–287, fig. 364–371; Vachon 1952a: 255–261, fig. 364–371; Foley 1951: 33; Bücherl 1964: 57; Pérez 1974: 22: l.evy and Amitai 1980: 15; El-Hennawy 1992: 98, 119; Kovařík 1995: 20; Kovařík 1998: 106; Fet and Lowe 2000: 92; Sousa et al. 2012: 68–69; Stockmann and Ythier 2010: 366–367; Aboumaâd et al. 2014: 6..
Buthus marocanus (ISS): Le Corroller 1967: 63.

#### Type material.

3 M, F, syntypes (ZIN), Morocco; 1 specimen syntype (ZIN), locality unknown (Fet and Lowe 2000).

#### Distribution.

all known specimens have been captured in the Rabat Region (approx. 28.43°, -11.10°).

#### Remarks.

it remains the only known *Buthus* species with a uniformly darkened body.

### 38. Buthus montanus

Taxon classificationAnimaliaScorpionesButhidae

Lourenço & Vachon, 2004

https://science.mnhn.fr/institution/mnhn/collection/rs/item/rs8604

https://science.mnhn.fr/institution/mnhn/collection/rs/item/rs8653

Buthus montanus Lourenço and Vachon 2004:84, 86–87, 91, fig. 16–30; Fernández 2004: 222; Fet 2010: 4; Rossi 2012: 274, 277–278; Teruel and Melic 2015: 5–9.

#### Type material.

1 M holotype (MNHN N° RS8604), Sierra Nevada (between Puerto de la Ragua and Cerro Pelado) (approx. 37.11°, -3.14°), Granada Region, Spain. Paratypes: 1 M, 3 F (MNHN N° RS8653), same locality.

#### Distribution.

known only from the type locality.

### 39. Buthus nigrovesiculosus

Taxon classificationAnimaliaScorpionesButhidae

Hirst, 1925 stat. n.

Buthus europaeus nigrovesiculosus : Hirst 1925: 416.
Buthus occitanus nigrovesiculosus : Pérez 1974: 22; Fet and Lowe 2000: 96.

#### Type material.

1 M (adult?), 1 juv., syntypes (NHMUK), Boste (approx. 23.79°, -15.68°), Rio de Oro (Western Sahara), now Morocco.

#### Distribution.

known only from the type locality.

#### Remarks.

**Figure 14.**
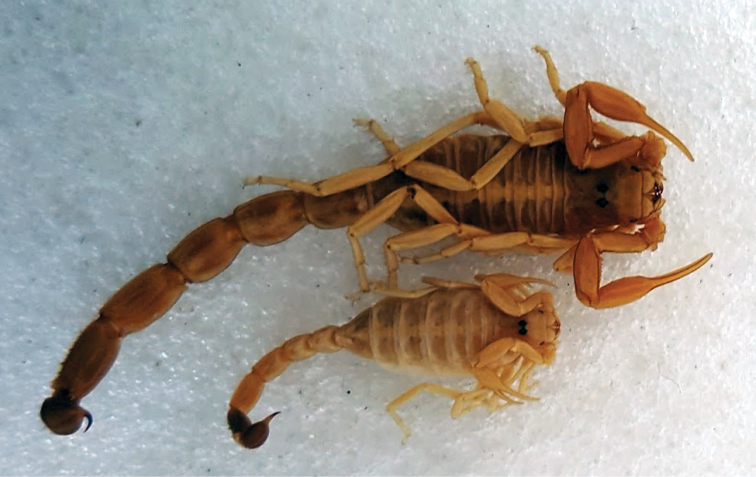
Photo of the syntypes of *B. nigrovesiculosus* (NHMUK). Photo by Sérgio Henriques.

**Figure 15.**
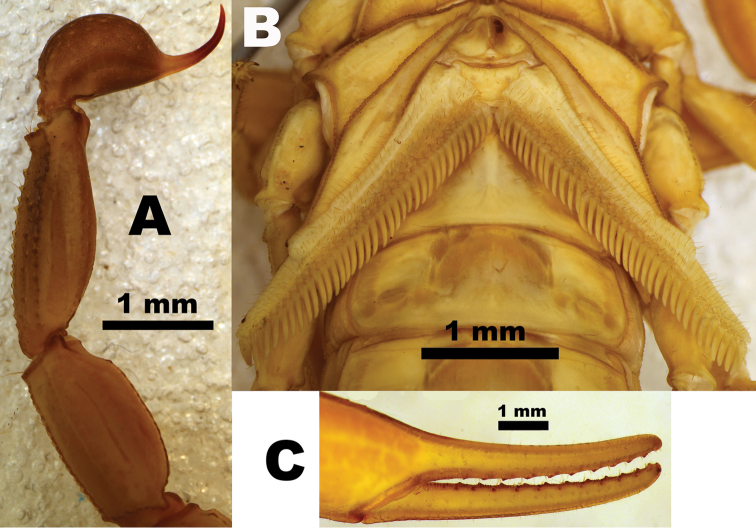
Detailed morphology of the larger syntype of *B. nigrovesiculosus* (NHMUK) **A** Lateral view of the telson and two terminal segments of the metasoma **B** Ventral view of the mesosoma, with pectines clearly visible **C** External lateral view of the terminal half of the right pedipalp che-la. All photos by Sérgio Henriques.

Hirst identified this North African species as a subspecies of *B. occitanus*, but as currently circumscribed, *B. occitanus* does not occur in North Africa (Gantenbein and Largiadèr 2003, Sousa et al. 2012). The original description of *B. nigrovesiculosus* suggests morphological similarities to *B. draa* and *B. tassili* Lourenço, 2002. These three species have a dark, almost black, fifth segment of the metasoma (Fig. 14 and 15A, less clear in the male type, obvious in the juvenile, syntypes in the NHMUK). Males of these three species also show slender pedipalp chelae. The type series of *B. nigrovesiculosus* includes only two animals, and more material is necessary to correctly evaluate the relationship between these three species. Nevertheless the males of *B. nigrovesiculosus* can be distinguished from males of the other two species by a higher pectinal tooth count (Fig. 10 and 15B, 36 versus <32 in the other two species), and from *B. tassili* by having a squared first metasomal segment.

### 40. Buthus occidentalis

Taxon classificationAnimaliaScorpionesButhidae

Lourenço, Sun & Zhu, 2009

https://science.mnhn.fr/institution/mnhn/collection/rs/item/rs8844

https://science.mnhn.fr/institution/mnhn/collection/rs/item/rs8845

Buthus occidentalis : Lourenço et al. 2009: 72–74, fig. 7–19; Yang et al. 2013: 2.

#### Type material.

1 F holotype (MNHN N° RS8844), Dakhlet Nouadhibou Region, in the coastal area (approx.20.28°, -16.24°), Mauritania. Paratypes: 1 M, 1 F juv. (MNHN N° 8845); 1 F, 1 M juv. (MHBU), all from the same locality.

#### Distribution.

known only from the type locality.

### 41. Buthus occitanus

Taxon classificationAnimaliaScorpionesButhidae

(Amoreux, 1789), restricted distribution

= Scorpio rufus: Amoreux 1789a: 42–43 (synonymized by Amoreux 1789b, as the first revisor, ICZN article 24.2.2).
= Androctonus ajax: C. L. Koch 1839b: 53, pl. CXCIII, fig. 467 (synonymized by Simon 1879: 96). Type lost: Spain.
= Androctonus eurialus: C. L. Koch 1839b: 25–27, pl. CLXXXVII (not pl. CXXXVIl, as listed in the text), fig. 448 (synonymized by Simon 1879: 96). Type lost, France.
= Androctonus eurilochus: C. L. Koch 1839b: 27–28, pl. CLXXXVII, fig. 449 (synonymized by Kraepelin 1891: 196). Type lost, locality unknown.
= Buthus europaeus: Thorell 1876b: 7. Type is the lost Linnaeus (1748, 1754), specimen, purportedly from Italy (see the above “type species of Buthus“ section and Fet et al. 2002).
= Buthus europaeus tridentatus: Franganillo 1918: 122–123 (**syn. n.**). Type unknown, Janvier, Navarre, Spain.
Scorpio occitanus : Amoreux 1789a: 42–43, pl. I, fig. 2; Amoreux 1789b: 10–16, pl. I. fig. 3; Herbst 1800: 73–82: Latreille 1804: 122; Latreille 1806: 132; Maccary 1810: 5–48; Audouin 1826: 172–173, pl. VIII, fig. 1; Audouin 1827: 410–411, pl. VIII, fig. 1; Dufour 1856 (part): 570.
Androctonus ajax : C. L. Koch 1850: 90.
Androctonus euryalus (ISS): C. L. Koch 1850: 90.
Androctonus eurylochus (ISS): C. L. Koch 1850: 90; Kraepelin 1891: 196.
Androctonus (Prionurus) occitanus : Lankester 1885: 380.
Buthus europaeus : Karsch 1879a: 18; Simon 1879: 96–98; Pavesi 1880: 312–313; Simon 1880c: 29; Pavesi 1884: 450; Pavesi 1885: 197, 199; Simon 1885: 51; Pocock 1889a: 116; Thorell 1893: 358–359; Birula 1896: 241–243; Birula 1900c: 9; Hirst 1925 (part): 415–416; Gadeau de Kerville 1926: 71; Bacelar 1928: 191; Hugues 1933: 487–488.
Buthus occitanicus (ISS): Dalla Torre 1905: 3; Táborský 1934: 40.
Buthus occitanus : Leach 1815: 391; Risso 1826: 156–157; Peters 1861b: 513; Karsch 1881a: 89; Kraepelin 1891 (part): 196–199 (part), pl. I, fig. 5, plI., fig. 18; Kraepelin 1895: 80; Kraepelin 1899: 26; Kraepelin 1901a (part): 266; Werner 1902 (part): 598; Birula 1910: 118–120; Masi 1912: 101; Borelli 1914b: 460; Lampe 1917: 191; Pavlovsky 1924: 77; Pavlovsky 1925: 140; Werner 1925: 209; Werner 1936: 173; Schenkel 1938: 4; Feytaud 1940: 38–39; Vachon 1940: 242–247, 254–258, fig. 1–9, 29, 33, 61–64; Käsmer 1941: 231; Denis 1948: 155–156; Vachon 1948c: 61, fig. 5; Vachon 1949a: 156–160, fig. 331–344, 348, 372–380; Vachon 1950b: fig. 591; Vachon 1951a: fig. 641, 657, 663, 679, 687, 696; Vachon 1951b: 621–623; Vachon 1952a: 264; Vachon 1952b: 274–279; Vachon 1961: 31–32; Bücherl 1964: 57; Pérez 1974: 22; Vachon 1974: 873; Goulliart 1979: 2; Levy and Amitai 1980 (part): 15–16; Kinzelbach 1982: 53; Prost 1982: 5; Mari et al. 1987; Sissom 1990: 92, fig. 3.17C, L; Kovařík 1992a (part): 183; Reichholf and Steinbach 1992: 33, fig. 4–5; Crucitti 1993: 51; Crucitti et al. 1994: 57–66; Vincent 1994: 6; Crucitti and Chinè 1995: 15–26; Braunwalder 1997b: 3; Crucitti and Chinè 1997: 195–200; Cloudsley-Thompson and Lourenço 1998: 1–2; Kovařík 1998: 106; Kovařík 1999 (part): 39, 42, fig. 3; Fet and Lowe 2000 (part): 92–94; Lourenço 2003 (part): 884, 886–887, fig. 27–32; Lourenço and Vachon 2004: 83–85, fig. 1–15; Kovařík and Whitman 2005 (part): 106; Teruel and Pérez-Bote 2005: 276; Castilla and Pons 2007: 258; Dupré et al. 2008: (pages unnumbered); Sousa et al. 2010: 207; Colombo 2011: 1; Rossi 2012: 274–278; Pedroso et al. 2013: 300; Rossi, Tropea and Yağmur 2013: 3; Martin-Eauclaire et al. 2014: 56; Teruel and Melic 2015: 6–9; Lourenço 2016b: fig. 2.
Buthus occitanus occitanus : Birula 1910: 118; Hadži 1929: 31; Vachon 1949a: 156–160, fig. 331–344; Vachon 1949c: 336; Vachon 1952a (part): 264; Le Corroller 1967: 63; Fet and Lowe 2000 (part): 94–95; Gantenbein and Largiadèr 2003 (part): 120, 122.
Buthus occitanus tridentatus : Fet and Lowe 2000: 97; Kovařík 2001: 79;
Buthus (Buthus) occitanus : Birula 1909b: 507; Birula 1910: 143; Birula 1914b: 644–664; Birula 1917a: 22, 38–39, 199, 213.
Buthus (Buthus) occitanus occitanus : Roewer 1943: 206.
Buthus cf. occitanus : Piñero et al. 2013: 88.
Scorpio australis (MIS): Asso 1784: 146, Tab. I, fig. 2.
Scorpio (Androctonus) occitanus : Gervais 1844a: 42–44; pl. XXIII, fig. 4.
Scorpio occitanicus (ISS): Serres 1822: 65.
*Scorpion Occitanus* (ISS): Latreille 1817: 105–106.

#### Type material.

type unknown, Souvignargues, Occitanie Region, France.

#### Distribution.

Traditionally, the distribution of *B. occitanus* was considered to span from the Moroccan Atlantic shores in North Africa to the Middle East in Asia and to Southern-Western Europe. However, following the description of new species in the genus, the present distribution of *B. occitanus* has been restricted to NE Spain and SW France. Several molecular phylogenetic studies have demonstrated that the species range does not extend beyond Western Europe (Gantenbein and Largiadèr 2003, Sousa et al. 2010, 2012, Pedroso et al. 2013). Lourenço and Vachon (2004) and Rossi (2012) include redescriptions of *B. occitanus* that include only European animals, but Vachon (1952a) also included material from Morocco, which was most likely not conspecific.

#### Remarks.

All material collected outside of the range here proposed should be considered as *Buthus sp*. Only the re-examination of those specimens could reveal their appropriate identity. Vachon (1952a) included animals from the west (Atlantic) coast of Morocco, from Kenitra to El Jadida, within his definition of *B. o. occitanus*. As explained above this material is now considered not to be part of *B. occitanus* and thus remains unnamed. We opted to leave *Androctonus eurilochus* in synonymy with *B. occitanus*, despite the uncertain about the species provenance, to help to stabilise the genus’ taxonomy. To further bring stability to *Buthus* taxonomy we propose Franganillo’s subspecies, *B. o. tridentatus*, as a junior synonym of *B. occitanus*. Although the type specimen is not known (if it ever existed as such), its type locality is clearly stated as Javier, in Navarre, Spain. Extensive sampling on the left bank of the Ebro River (Sousa 2017) indicates that only *B. occitanus* occurs in this part of Spain and hence we here propose this new synonymy.

### 42. Buthus orientalis

Taxon classificationAnimaliaScorpionesButhidae

Lourenço & Simon, 2012

http://zoobank.org/E408579C-9287-4EE7-9C32-E754EC925B92

https://science.mnhn.fr/institution/mnhn/collection/rs/item/rs8910

https://science.mnhn.fr/institution/mnhn/collection/rs/item/rs6623

Buthus orientalis : Lourenço and Simon 2012: 10–14, fig. 1–12; Rossi 2013: 191–192; Rossi, Tropea and Yağmur 2013: 5, 7.

#### Type material.

1 F holotype (MNHN N° RS8910), Alexandria (approx. 31.17°, 29.91°), Egypt. Paratypes: 7 M, 13 F (MNHN, N° RS6623), same locality.

#### Distribution.

known only from the type locality.

### 43. Buthus paris

Taxon classificationAnimaliaScorpionesButhidae

(C. L. Koch, 1839)

= Androctonus clytoneus: C. L. Koch 1839a: 70–72, pl. CLXIII, fig. 384 (synonymized by Vachon 1949c: 380–381). Types lost; Africa.
Androctonus paris : C. L. Koch 1839a: 25–28, pl. CLI, fig. 352; C. L. Koch 1850: 90.
Androctonus clytonicus (ISS): Gervais 1844a: 43.
Androctonus clytoneus : C. L. Koch 1850: 90.
Buthus occitanus paris : Birula 1903: 107; Birula 1910: 118, 155; Giltay 1929: 196; Werner 1932: 300–305; Vachon 1949c: 380–388, fig. 356, 400, 445–455; Vachon 1951b: 621; Vachon 1952a: 308–316, fig. 356, 400 445–455; Malhomme 1954: 29; Arroyo 1961: 186–189; Le Corroller 1967: 63; Peréz 1974: 23; Levy and Amitai 1980: 16; El-Hennawy 1992: 98, 121; Kovařík 1995: 20; Gantenbein et al. 1998a: 51; Kovařík 1998: 106; Fet and Lowe 2000: 96; Touloun et al. 2001: 2; Gantenbein and Largiadèr 2003: 120, 122; Touloun 2012: 35, 104, 108, fig.5B.
Buthus (Buthus) occitanus paris : Birula 1910. 145, 155; Birula 1917a: 223.
Buthus paris : Lourenço 2003: 896–897, fig. 52–56; Kovařík 2006: 2, 6, 8, 15, fig. 10–11; Lourenço 2013: 65–66; Rossi, Tropea and Yağmur 2013: 3, 5, 7; Aboumaâd et al. 2014: 6; Touloun et al. 2014: 77–78; Lourenço and Sadine 2016: 14–15.

#### Type material.

Holotype lost according to Fet and Lowe (2000), Algeria. Vachon (1949c, 1952a) wrote that the types came from Alger without further explanation.

#### Distribution.

the species is currently distributed across Algeria, Morocco and Tunisia.

#### Remarks.

Because of the description of new species from Algeria, and the lack of both type specimen and locality (beyond the country), a neotype for *B. paris* is necessary to stabilize the taxonomy of Algerian *Buthus*, which may challenge the status of some newly described species. This is further complicated by recent diagnoses of *B. paris* (at least in part: Lourenço 2003, Kovařík 2006, Lourenço and Sadine 2016) that differ from those offered by Vachon (1952a). The differences between the different diagnoses include the number of rows in the movable finger, the aspect ratio of the first metasomal segment, the body chaetotaxie, the aculeus to vesicle length and the type of sexual dimorphism of the pedipalp chelae. Vachon (1952a) studied a large number of specimens from the entire Maghreb region, unrivalled by any subsequent study, which leads us to consider Vachon’s description as the “gold standard”. Vachon himself stated that most of the specimens used in his redescriptions were stored at the MNHN. If this material is ever located, it should have priority in the designation of a neotype. Although Vachon (1952a) did not formally describe any varieties within *B. paris*, he split the specimens that compose the species into three regions: 1) The typical region (from Algiers to northern Tunisia); 2) Specimens from the Oujda region (Morocco); 3) Those from northern Morocco and the Middle Atlas flanks. Interestingly the split of *B. paris* into these three regions corresponds well with the distribution of the genetic variability in the *cox1* gene (Gantenbein and Largiadèr 2003, Sousa et al. 2012, Pedroso et al. 2013), placing *B. paris* in two different groups: in *occitanus* which include all *B. paris* specimens from regions two and three, and *tunetanus*, which include *B. paris* specimens from the typical region one, along the split of the two *cox1* groups in the middle of Algeria (Fig. 7). If confirmed, this will mean that *B. paris* does not occur in Morocco. It is unclear if the variety from the third region above might correspond to *B. confluens* Lourenço, Touloun & Boumezzough, 2012, although these authors (page 22) refrained from suggesting this possibility because they could not find any of the material used by Vachon, purportedly to be in the MNHN, to describe this variety.

### 44. Buthus parroti

Taxon classificationAnimaliaScorpionesButhidae

Vachon, 1949 stat. n.

Buthus atlantis parroti : Vachon 1949a: 168–169, fig. 346, 350, 352–354, 356, 363; Vachon 1952a: 254–255, fig. 346, 350, 352, 353, 354, 356, 363; Le Corroller 1967: 63; Pérez, 1974 1974: 22; El-Hennawy 1992: 98, 119; Kovařík 1995: 20; Kovařík 1998: 106; Lourenço 2003: 883–885, fig. 23–26; Fet and Lowe 2000: 91–92; Touloun 2012: 43, fig. 9B; Pedroso et al. 2013: 300.

#### Type material.

1 F (MNHN N° RS1870), 1F, 12 juv., syntypes (MNHN), Forest house (approx. 30.31°, -9.33°), Ademine Forest, 40 Km S.W. of Agadir, Morocco; 2 M, 1 F juv., syntypes (MNHN), Taroudant (approx. 30.46°, -8.87°), Morocco.

#### Distribution.

Known only from the Sous River Valley. Type localities in Fet and Lowe (2000) were mixed up.

#### Remarks.

**Figure 16.**
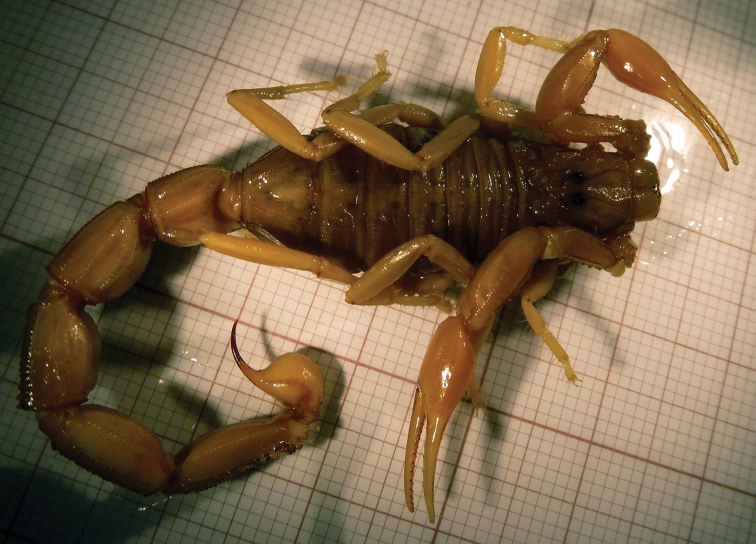
Photo of a *B. parroti* female syntype (MNHN Nº RS1870), from the Ademine Forest, 04-1939, S.W. of Agadir, Morocco (Vachon 1952b).

*B. parroti* was first described as a subspecies of *B. atlantis*. The two species occupy different habitats in Western Morocco; *B. parroti* is a forest species and *B. atlantis* is a sand dune dweller (Vachon 1952a). Furthermore, *B. atlantis* is clearly larger than *B. parroti*. Additionaly, the first metasomal segment of *B. parroti* is wider than long, the aculeus is shorter than the vesicle and the anal arch has only two lobes (Vachon 1952a), while in *B. atlantis* this segment is longer than wide, the aculeus is as long as or longer than the vesicle and the anal arch has three lobes (Vachon 1952a). Three additional *Buthus* species occur in the same area of Morocco as *B. parroti*, namely *B. elmoutaouakili* Lourenço & Qi, 2006, *B. mardochei* Simon, 1878 and *B. mariefranceae*. *B. parroti* can be distinguished from all three species by the presence of macrosetae in the terguites (Vachon 1952a). Moreover, it can be distinguished from *B. mariefranceae* by its larger size and absence of a dark fifth metasomal segment. Mesosoma colour pattern is not clear for *B. parroti*; the examined specimen at the MNHN (RS1870) (Fig. 16) appears to have two very faint darker stripes, while *B. mariefranceae* has two very well marked mesosomal dark stripes. *B. parroti* males show slender pedipalp chelae than females, while there is little if any sexual dimorphism in *B. mardochei*.

### 45. Buthus prudenti

Taxon classificationAnimaliaScorpionesButhidae

Lourenço & Leguin, 2012

https://science.mnhn.fr/institution/mnhn/collection/rs/item/rs8913

https://science.mnhn.fr/institution/mnhn/collection/rs/item/rs8915

https://science.mnhn.fr/institution/mnhn/collection/rs/item/rs8914

Buthus prudenti : Lourenço and Leguin 2012: 2–6, 8, fig. 1–14; Lourenço 2016a: 76.

#### Type material.

1 M holotype (MNHN N° RS8913), Ouro Labaré (9.38715°, 13.83447°), Bénoué, Cameroon. Paratypes: 7 M, 8 F, same locality; 11 paratypes (MNHN N° RS8914, RS8915), 4 paratypes (CBGP).

#### Distribution.

known only from the type locality.

#### Remarks.

type locality toponym derived from the coordinates given in the original description, as the type locality given was only Region of Sanguéré-Djoi, Cameroon.

### 46. Buthus pusillus

Taxon classificationAnimaliaScorpionesButhidae

Lourenço, 2013

Buthus pusillus : Lourenço 2013: 64–67, fig. 1–9; Lourenço and Sadine 2016: 14; Rossi, Tropea and Yağmur 2013: 5, 7.

#### Type material.

1 M holotype (ZMH N° A11/13), Tizi Oumalou (36.5102°, 4.3390°), Tizi Ouzou Province, Djurdjura Mountains, Algeria. Paratype: 1 M juv. (ZMH N° A12/13), same location.

#### Distribution.

known only from the type locality.

#### Remarks.

The locality we present here corresponds to the coordinates given in the paper, although these do not agree with the elevation also reported in the paper: 2150 m a.s.l. This is very close to the maximum altitude of the highest peaks of the Djurdjura Mountains, and much higher than the 935 m a.s.l. of Tizi Oumalou.

### 47. Buthus rochati

Taxon classificationAnimaliaScorpionesButhidae

Lourenço, 2003

Buthus rochati : Lourenço 2003: 893–896, fig. 47–51;Aboumaâd et al. 2014: 6.
Buthus occitanus mardochei panousei : Vachon 1949c: 373–376, fig. 426–432; Vachon 1952a: 301–304, fig. 426–432; Le Corroller 1967: 63; Pérez 1974: 23; Touloun 2012: 57.

#### Type material.

1 M holotype (MHNG), Tafnidilt Region (as Tafnidit) (approx. 28.56°, -11.03°), Guelmim Region, Morocco. Paratypes: 1 M, 1 F (MHNG); same locality; 6 F (MHNG), west of Tafnidilt Region, Draa River valley; 3 M, 1 F (MHNG), unknown locality.

#### Distribution.

known distribution confined to the Tafnidilt region of Morocco.

#### Remarks.

Lourenço (2003) does not mention the most remarkable diagnostic character given by Vachon (1952a), the interrupted dorso-median keel of the pedipalp patella. However Lourenço's Figure 49 illustrates this character, as it is a copy of Vachon's original drawings, and thus we consider it as part of the species diagnose and the most reliable diagnostic character for *B. rochati*. Vachon (1949) infrasubspecific name is not available as is explained above.

### 48. Buthus saharicus

Taxon classificationAnimaliaScorpionesButhidae

Saddine, Bissati & Lourenço, 2015

Buthus saharicus : Saddine et al. 2015: 47–49, fig. 6–8; Lourenço 2016b: fig. 4.

#### Type material.

1 F holotype (MNHN), Ghardaïa Region (approx. 32.300°, 3.833°), in Wadi bed, Algeria. Paratypes: 1 M (UGA), 1 F juv. (MNHN), same locality.

#### Distribution.

known only from the type locality.

#### Remarks.

Saddine et al. (2015) claimed that *B. saharicus* was the “first true deserticolous species found in Algeria”, a bold claim given that *B. tunetanus* (*sensu* Vachon 1949, part) had already been recorded for Beni Abbés by Vachon (1949, 1952), also a desert location in central west Algeria, and albeit neither localities are Erg Desert areas, Beni Abbés is in the border of the Grand Erg Occidental.

### 49. Buthus tassili

Taxon classificationAnimaliaScorpionesButhidae

Lourenço, 2002

https://science.mnhn.fr/institution/mnhn/collection/rs/item/rs8501

https://science.mnhn.fr/institution/mnhn/collection/rs/item/rs8622

Buthus tassili : Lourenço 2002: 113–115, fig. 10, 12, 14; Lourenço 2003: 906–909, fig. 80–86; Rossi, Tropea and Yağmur 2013: 3–5, 7.
Buthus occitanus (MIS): Pallary 1929: 134, 140; Pallary 1934: 98–99.
Buthus occitanus tunetanus neeli : Gysin 1969: 65–71, fig. 1–5; Peréz 1974: 22; Touloun 2012: 37, 40.
Buthus occitanus tunetatus , “Spécimens des régions montagneuses centrales du Sahara”: Vachon 1952a: 279.

#### Type material.

1 M holotype (MNHN N° RS8501), Tin Tazarif (approx. 24.466°, 10.466°), Illizi, Algeria. Paratype: 1 F (MNHN N° RS8622), same locality.

#### Distribution.

This species is known from a wide area around the Hoggar and Tassili N’Ajjer Mountains, including at least one locality in Libya.

#### Remarks.

The Tin Tazarif coordinates given here, standing at 880 m a.s.l., do not match the altitude given for the point by Lourenço (2002), of 1.800 m a.s.l., but correspond well to the map location given by the author in Figure 6 of the same article. Nevertheless there are several locations in the Tassili N’Ajjer Mountains at or above 1.800 m a.s.l., located closer to Jebel Azao, its highest peak. The species ranges from the Hoggar Mountains to the Tassili N’Ajjer Mountains, including Ghat in Libya. Gysin’s name, *B. o. t. neeli*, is not available under the ICZN article 10.2 and 45.5, as already stated by Fet and Lowe (2000). Therefore, it cannot enter formal synonymy. Nevertheless, because Gysin’s description (1969) brings relevant taxonomic information (figures and new localities) for *B. tassili*, we have decided to clearly state this new informal synonymy. The specimens studied of both species come from the Algerian Hoggar Mountains and share a typical darkened fifth segment of the metasoma (see *B. nigrovesiculosus* above).

### 50. Buthus trinacrius

Taxon classificationAnimaliaScorpionesButhidae

Lourenço & Rossi, 2013

Buthus trinacrius : Lourenço and Rossi 2013: 10–12, fig. 1–9.
Buthus europaeus (MIS): Simon 1879: 97; Simon 1910 (part): 69.
Buthus occitanus (MIS): Kraepelin (1901): 266.

#### Type material.

1 M holotype, Palermo Province? (approx. 38.05°, 13.32°), Sicily. Paratypes: 1 M, 1 F. All type material in bad conditions (MNHN N° RS3247).

#### Distribution.

Recorded from Sicily.

#### Remarks.

Although Lourenço and Rossi (2013) report that the collector is not mentioned in Simon's notes, Kraepelin (1901), in his list of all the scorpion material present in the MNHN Paris, writes that Letourneur collected the *Buthus* material from Sicily and Corfu (Greece), which causes doubts regarding the correct collection locality of the specimens used to describe this species. Simon (1879) had doubts about the actual existence of *Buthus* in Sicily (when examining the specimens that would eventually be designated as type material for *B. trinacrius*): “*il habite probablement aussi le midi de l’Italie et la Sicile*”, and *Buthus* has never been found in mainland Italy. Furthermore, the authors also claimed that Simon (1910) “referred to the almost impossibility to distinguish *Buthus* populations from North of Africa with those from Spain and Sicily”, which is only partially correct. Simon (1910) solely refered to the distinction of *Buthus* populations of Algeria and Spain from those of Egypt, although in a subsequent paper, Simon does state that *Buthus* exist in Sicily without any further comments.

### 51. Buthus tunetatus

Taxon classificationAnimaliaScorpionesButhidae

(Herbst, 1800)

Scorpio tunetanus Herbst 1800: 68–69, pl. III, fig. 3 (not pl. II, fig. 2, as listed in the text); Latreille 1804: 122–124.
Androctonus (Leiurus) tunetanus : Ehrenberg in Hemprich and Ehrenberg 1829: 354.
Androctonus (Leiurus) tunetanus genuinus : Ehrenberg in Hemprich and Ehrenberg 1829: 354.
Androctonus (Liurus) tunetanus : Ehrenberg in Hemprich and Ehrenberg 1831 (pages unnumbered)
Androctonus (Liurus) tunetanus genuinus : Ehrenberg in Hemprich and Ehrenberg 1831 (pages unnumbered).
Androctonus tunetanus : C. L. Koch 1845: 15–19, pl. CCCCI (sic), fig. 968; C. L. Koch 1850: 90.
Buthus occitanus (MIS): Kovařík and Whitman 2005 (part): 106.
Buthus occitanus tunetanus : Birula 1903: 107; Birula 1910: 118; Borelli 1914a: 154–155; Borelli 1914b: 461; Borelli 1924: 4–5; Borelli 1928: 351; Giltay 1929: 196–197; Werner 1929: 30–31; Caporiacco 1932: 395–396; Schenkel 1932: 379–380; Werner 1932: 300–305; Pallary 1934: 99; Borelli 1934: 169; Werner 1934b: 84–85, fig. 4; Werner 1936: 173; Caporiacco 1937: 345; Schenkel 1949: 186; Vachon 1949c: 344–353, fig. 381–393; Vachon 1951a: fig. 670; Vachon 1952a: 272–281, fig. 381–393, 670; Vachon 1966: 211; Peréz 1974: 22; Levy and Amitai 1980: 16; El-Hennawy 1992: 98, 121; Kovařík 1995: 20; Kovařík 1997: 179; Gantenbein et al. 1998a: 51; Gantenbein et al. 1998b: 33–39; Kovařík 1998: 106; Fet and Lowe 2000: 97; Lourenço 2002, p. 113, 115, fig. 8–9, 11, 13; Kovařík 2002: 6; Gantenbein and Largiadèr 2003: 120, 122; Ben Othmen et al. 2004: 257; Touloun 2012: 37, 41.
Buthus (Buthus) occitanus tunetanus : Birula 1908: 123–124; Birula 1909: 507–508, fig. B; Birula 1910: 156–157; Birula 1917a: 223; Roewer 1943: 206.
Buthus tunetanus : Simon 1872: 251–252; Lourenço 2003: 897–899, fig. 57–61; Kovařík 2006: 2, 8, 10, 15, fig. 16–19.; Sadine et al. 2011: 6; Lourenço and Cloudsley-Thompson 2012: 13–16, fig. 8; Lourenço and Simon 2012: 12; Lourenço 2013: 65–66; Rossi, Tropea and Yağmur 2013: 4–5, 7
*Scorpion Tunetanus* (ISS): Latreille 1817: 106.

#### Type material.

Types lost according to Fet and Lowe (2000), Tunisia.

#### Distribution.

The species is currently distributed across Algeria, Libya, Morocco and Tunisia, and doubtfully in the island of Malta.

#### Remarks.

Because of the description of new species from Tunisia, and the lack of both type specimen and locality (beyond the country), a neotype for *B. tunetanus* is necessary to stabilize the taxonomy of Tunisian *Buthus*. As explained for *B. paris*, this is further complicated by recent diagnoses of *B. tunetanus* that differ from those offered by Vachon (1952a), and as such if Vachon’s *B. tuntetanus* material is found in the MNHN it should be given priority in the future designation of a neotype. Vachon (1952a) did not formally described any variety of *B. tunetanus*, but he again split the specimens that compose the species into four regions: 1) the typical region, corresponding to north and central Tunisia; 2) the southern montane region of Algeria, specimens from which have subsequently been described as *B. tassili*; 3) the Algerian Saharan Atlas and the southern region of the High Plateau; and 4) the disjunct desert regions of southern Tunisia, western central Algeria and eastern central Morocco. It is unclear whether region 3 or 4 might either correspond to *B. dunlopi* or *B. saharicus*. As explained in Fet and Lowe (2000), the name A. (Leiurus) t. genuinus refers to the nominotypical form of the species and as such the adjective “genuinus” is not an available subspecific name.

### 52. Buthus yemenensis

Taxon classificationAnimaliaScorpionesButhidae

Lourenço, 2008

Buthus yemenensis : Lourenço 2008: 47–50, fig. 1–7.

#### Type material.

1 F holotype (ZMH N° A33/08), Ma'bar (approx. 14.8°, 44.3°), Dhamar, Yemen.

#### Distribution.

Known only from the type locality.

### 53. Buthus

Taxon classificationAnimaliaScorpionesButhidae

sp.

Buthus albengai (MIS): Habel et al. 2012: 3–4.
Buthus europaeus (MIS): Simon 1899: 85; Simon 1910 (part): 68–70, fig. 5, 8.
Buthus malhommei (MIS): Habel et al. 2012: 3–4.
Buthus occitanus (MIS): Karsch 1881b: 8 (Libya); Pocock 1899: 834 (Africa); Kraepelin 1901a (part): 266; Werner 1902 (part): 598; Chaignon 1904: 83–84 (Tunisia); Tullgren 1909: 2–3 (Egypt); Borelli 1924 (Libya): 4; King 1925: 81 (Sudan); Gough and Hirs 1927: 5, fig. 9 (Egypt); Pallary 1934: 98–99; Werner 1934a: 269, fig. 330 (Morocco); Pallary 1938: 281–282; Sergent 1938: 519–520, pl. 49; Monard 1939: 82–83 (Guinea-Bissau); Moriggi 1941: 84; Sergent 1941a: 355, fig. 1E, 2.7, pl. 35, fig. 7; Sergent 1941b: 447, plate 37; Vachon 1941: 52; Frade 1947: 268 (Guinea-Bissau); Vachon 1952a (part): 262–271, fig. 331–344, 348, 372–380, 591, 641, 657, 663, 679, 687, 696; Vachon 1953: 1021–1024, fig. 12 (Mauritania); Malhomme 1954: 28 (Morocco); Belfield 1956: 44; Kinzelbach 1975: 14, fig. 1; Lamoral and Reynders 1975: 505 (Africa); Levy and Amitai 1980 (part): 15–16; Kinzelbach 1984: 100 (Asia); Kinzelbach 1985: map II (Asia); El-Hennawy 1987: 17 (Egypt); Amr et al. 1988: 374 (Jordan); Michalis and Dolkeras 1989: 265–266 (Greece); El-Hennawy 1992: 98, 101, 119–120 (Arabia); Kovařík 1992a (part): 183; Kovařík 1992b: 90 (Iraq); Amr and EI-Oran 1994: 181 (Jordan); Kovařík 1997: 179 (Maghreb); Fet and Lowe 2000 (part): 92–94; Kovařík 2002: 5; Lourenço 2003: 884 (Morocco) Soleglad and Fet 2003: 7 (Morocco); Kaltsas et al. 2008: 215–216 (Libya); Sadine et al. 2012: 33; El-Hennawy 2013: 260; Aboumaâd et al. 2014: 5.
Buthus occitanus occitanus (MIS): Pocock 1895: 299 (Egypt); Vachon 1952a (part): 262–271, fig. 331–344, 372–379, 400, 554; Levy and Amitai 1980: 16 (Africa); Kovařík 1995: 20 (Morocco); Kovařík 1997: 179 (Ghana); Gantenbein et al. 1998a: 51, Fet and Lowe 2000 (part): 94–95; Gantenbein and Largiadèr 2003 (part): 120, 122.
Buthus occitanus occitanus , “Afrique occidentale française”: Vachon 1952a: 270.
Buthus occitanus occitanus , “Cote Occidentale du Maroc”: Vachon 1952a: 268–269, fig. 373–378.
Scorpio occitanus : Dufour 1856 (part): 570.
not Buthus: Vachon 1955: 372 (El Fâcher, Djebel Meidob, Darfur, Sudan) (Vachon said that this material was close to but not part of the genus Buthus).

#### Note.

We futher propose to transfer a species from the genus *Buthus* to the genus *Androctonus*.

### Androctonus barbouri

Taxon classificationAnimaliaScorpionesButhidae

(Werner, 1932) comb. n.

Buthus barbouri : Werner 1932: 300, fig. 141; Vachon 1949b: 287–288, fig. 371; Vachon 1952a: 261–262; Perez 1974: 22; El-Hennawy 1992: 98, 119; Kovařík 1995: 20; Kovařík 1998: 106; Fet and Lowe 2000: 92.

#### Type material.

1 M (type probably lost), Agadir (approx. 30.43°, -9.60°), Morocco.

#### Distribution.

known only from the type locality, Agadir, just north of the Sous River mouth, in southern Morocco.

#### Remarks.

The species *B. barbouri* was described by Werner from Agadir, Morocco. However, this species cannot be linked to any of the *Buthus* species known from the reported type locality. This problem was already recognized by Vachon (1952a) that considered this species to have been “imperfectly described”, but then failed to make any taxonomic or nomenclatural act to fix it. *Buthus barbouri* and *B. marrocanus* are the only fully dark *Buthus* species known from Morocco. *B. marrocanus* was described from the Rabat region, 500 km distant from Agadir, and is distinguished from the former species by the shape of the inferior lateral keels of the fifth segment of the metasoma and by the number of pectinal teeth (Vachon, 1952a). Because of the chaetotaxy of the pedipalps Vachon (1952a) considered *B. barbouri* to be closer to *Androctonus mauritanicus* (Pocock, 1902). In agreement with these diagnostic characters, and Vachon’s (1952) opinion, we transfer *B. barbouri* to the genus *Androctonus*, with the new combination *Androctonus barbouri* (Werner, 1932). The relationship between *A. barbouri* and *A. mauritanicus bourdoni* Vachon, 1948, a subspecies of *A. mauritanicus* (Pocock, 1902), another black *Androctonus* species that also occurs in the Sous River valley, should be investigated.

## Supplementary Material

XML Treatment for Buthus

XML Treatment for Buthus adrianae

XML Treatment for Buthus albengai

XML Treatment for Buthus amri

XML Treatment for Buthus atlantis

XML Treatment for Buthus aures

XML Treatment for Buthus awashensis

XML Treatment for Buthus barcaeus

XML Treatment for Buthus berberensis

XML Treatment for Buthus bonito

XML Treatment for Buthus boumalenii

XML Treatment for Buthus brignolii

XML Treatment for Buthus centroafricanus

XML Treatment for Buthus chambiensis

XML Treatment for Buthus confluens

XML Treatment for Buthus draa

XML Treatment for Buthus dunlopi

XML Treatment for Buthus duprei

XML Treatment for Buthus egyptiensis

XML Treatment for Buthus elhennawyi

XML Treatment for Buthus elizabethae

XML Treatment for Buthus elmoutaouakili

XML Treatment for Buthus elongatus

XML Treatment for Buthus hassanini

XML Treatment for Buthus ibericus

XML Treatment for Buthus intermedius

XML Treatment for Buthus intumescens

XML Treatment for Buthus israelis

XML Treatment for Buthus jianxinae

XML Treatment for Buthus karoraensis

XML Treatment for Buthus kunti

XML Treatment for Buthus labuschagnei

XML Treatment for Buthus lienhardi

XML Treatment for Buthus lourencoi

XML Treatment for Buthus malhommei

XML Treatment for Buthus mardochei

XML Treatment for Buthus mariefranceae

XML Treatment for Buthus maroccanus

XML Treatment for Buthus montanus

XML Treatment for Buthus nigrovesiculosus

XML Treatment for Buthus occidentalis

XML Treatment for Buthus occitanus

XML Treatment for Buthus orientalis

XML Treatment for Buthus paris

XML Treatment for Buthus parroti

XML Treatment for Buthus prudenti

XML Treatment for Buthus pusillus

XML Treatment for Buthus rochati

XML Treatment for Buthus saharicus

XML Treatment for Buthus tassili

XML Treatment for Buthus trinacrius

XML Treatment for Buthus tunetatus

XML Treatment for Buthus yemenensis

XML Treatment for Buthus

XML Treatment for Androctonus barbouri
